# Ancestral Genes Can Control the Ability of Horizontally Acquired Loci to Confer New Traits

**DOI:** 10.1371/journal.pgen.1002184

**Published:** 2011-07-21

**Authors:** H. Deborah Chen, Mollie W. Jewett, Eduardo A. Groisman

**Affiliations:** 1 Department of Molecular Microbiology, Washington University School of Medicine, Saint Louis, Missouri, United States of America; 2 Howard Hughes Medical Institute, Washington University School of Medicine, Saint Louis, Missouri, United States of America; Universidad de Sevilla, Spain

## Abstract

Horizontally acquired genes typically function as autonomous units conferring new abilities when introduced into different species. However, we reasoned that proteins preexisting in an organism might constrain the functionality of a horizontally acquired gene product if it operates on an ancestral pathway. Here, we determine how the horizontally acquired *pmrD* gene product activates the ancestral PmrA/PmrB two-component system in *Salmonella enterica* but not in the closely related bacterium *Escherichia coli*. The *Salmonella* PmrD protein binds to the phosphorylated PmrA protein (PmrA-P), protecting it from dephosphorylation by the PmrB protein. This results in transcription of PmrA-dependent genes, including those conferring polymyxin B resistance. We now report that the *E. coli* PmrD protein can activate the PmrA/PmrB system in *Salmonella* even though it cannot do it in *E. coli*, suggesting that these two species differ in an additional component controlling PmrA-P levels. We establish that the *E. coli* PmrB displays higher phosphatase activity towards PmrA-P than the *Salmonella* PmrB, and we identified a PmrB subdomain responsible for this property. Replacement of the *E. coli pmrB* gene with the *Salmonella* homolog was sufficient to render *E. coli* resistant to polymyxin B under PmrD-inducing conditions. Our findings provide a singular example whereby quantitative differences in the biochemical activities of orthologous ancestral proteins dictate the ability of a horizontally acquired gene product to confer species-specific traits. And they suggest that horizontally acquired genes can potentiate selection at ancestral loci.

## Author Summary

The traits that distinguish closely related bacterial species are often ascribed to differences in gene content, which arise primarily through horizontal gene transfer. In some instances, the genes mediating a new trait act as independent entities that function in a variety of organisms. However, the ability of a horizontally acquired gene product(s) to operate on an ancestral pathway might be constrained by subtle differences between orthologous ancestral proteins. Here, we examine why the horizontally acquired *pmrD* gene product post-translationally activates the ancestral PmrA/PmrB two-component system in *Salmonella enterica* but not in the closely related species *Escherichia coli*. This allows *Salmonella*, but not *E. coli*, to transcribe PmrA-activated genes including those conferring antibiotic resistance when grown in low Mg^2+^, which is a condition that promotes PmrD expression. We now demonstrate that, paradoxically, the *E. coli* PmrD protein activates the PmrA/PmrB system in *Salmonella* even though it fails to do so in *E. coli*. We establish that quantitative differences in the biochemical activities of the PmrB proteins from *Salmonella* and *E. coli* dictate the functionality of PmrD, which protects phosphorylated PmrA from PmrB's phosphatase activity. Our findings demonstrate that ancestral genes can control the ability of horizontally acquired genes to confer species-specific traits upon different organisms.

## Introduction

Closely related bacterial species often exhibit significant differences in gene content [1]. Whereas these differences can arise from the duplication and divergence or the loss of ancestral genes [2]–[4], the vast majority of species-specific DNA sequences in bacteria appear to have arisen as a result of horizontal gene transfer events [1], [5]–[7]. Species-specific sequences often endow bacteria with new abilities, which enable them to access unexplored niches such as animal and plant tissues [1], [8]. For example, some genomic islands from pathogenic bacteria function as freestanding virulence “cassettes”, and thus, the pathogenic properties they specify can be “played” effectively when the genomic island is introduced into a different bacterial species, even a non-pathogenic one [9]. Still, other horizontally acquired genes operate on ancestral cellular pathways [10], [11], raising questions as to the changes experienced both by the horizontally acquired DNA and the host bacterial genome that enable an organism to realize the fitness gains mediated by the acquired sequences.

This question can be addressed by first identifying a horizontally acquired gene product that behaves differently in present-day bacterial species, and by subsequently determining the changes in the horizontally acquired locus and/or associated ancestral genes that are responsible for a change in function. Such a strategy can provide insight into the evolutionary events that yielded extant diversity. Here, we provide a singular example of how an ancestral locus controls the ability of a horizontally acquired gene to confer a new ability.

**Figure 1:**
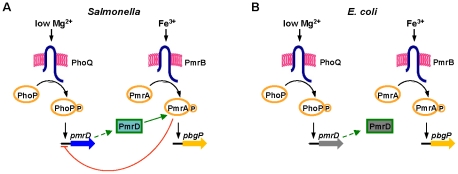
Model of the interactions between the PhoP/PhoQ and PmrA/PmrB two-component systems in *Salmonella* and *E. coli*. (A) In wild-type *Salmonella*, transcription of PmrA-activated genes is promoted during growth in low Mg^2+^ via the PhoP/PhoQ system, the PmrD protein and the PmrA/PmrB system, and in the presence of Fe^3+^ via the PmrA/PmrB system and independently of PhoP/PhoQ and PmrD. The PmrA protein represses transcription of the *pmrD* gene. (B) In wild-type *E. coli*, transcription of PmrA-activated genes is promoted in the presence of Fe^3+^ via the PmrA/PmrB system. The PmrD protein is produced in low Mg^2+^ in a PhoP-dependent manner but fails to activate the PmrA/PmrB system.

The *pmrD* gene product enables *Salmonella enterica* serovar Typhimurium to express loci governing modifications of the lipopolysaccharide (LPS) [12], rendering the organism resistant to killing by the antibiotic polymyxin B (Figure 1A) [13]–[16]. Transcription of these loci is controlled by the PmrA/PmrB two-component regulatory system [13], [17]–[19]. The PhoP-activated *pmrD* gene is expressed when *Salmonella* experience low levels of extracytoplasmic Mg^2+^, which is an inducing signal for the PhoP/PhoQ two-component system [20]. The PmrD protein binds to the phosphorylated form of the DNA binding protein PmrA (PmrA-P), protecting it from dephosphorylation by the sensor PmrB; this enhances PmrA-P levels and promotes transcription of PmrA-dependent genes [21]. Thus, *Salmonella* can resist killing by polymyxin B not only in response to the Fe^3+^ signal sensed by PmrB [22] but also in low Mg^2+^ environments [12].

The closely related species *Escherichia coli* induces expression of PmrA-regulated genes and resistance to polymyxin B following growth in the presence of Fe^3+^ (Figure 1B) [14], [16], [23]. However, *E. coli* does not activate the PmrA/PmrB system in low Mg^2+^ conditions even though its *pmrD* gene is expressed in a PhoP/PhoQ-dependent manner [23]. The inability of *E. coli* to activate the PmrA/PmrB system, and thus, to resist killing by polymyxin B in low Mg^2+^, has been attributed to its PmrD protein being highly divergent from the *Salmonella* PmrD [23]. Indeed, replacement of the *E. coli pmrD* gene by the *Salmonella* homolog enabled activation of the *E. coli* PmrA/PmrB system in low Mg^2+^ [23].

We now show how an ancestral protein can control the ability of a horizontally acquired gene product to confer new traits. We present evidence suggesting that *pmrD* was acquired by lateral transfer. We demonstrate that the highly divergent *E. coli pmrD* gene can rescue a *Salmonella pmrD* mutant to activate the PmrA/PmrB system during growth in low Mg^2+^. Beyond reinforcing the notion that the *E. coli pmrD* gene specifies a functional protein [24], our findings indicate that *Salmonella* and *E. coli* must differ in an additional component controlling the levels of PmrA-P. We establish that this component is PmrB as the *E. coli* PmrB exhibits significantly higher phosphatase activity than the *Salmonella* homolog, and that replacement of the *E. coli pmrB* gene with the *Salmonella* homolog is sufficient to restore transcription of PmrA-activated genes and polymyxin B resistance to *E. coli* grown in low Mg2+. Our findings provide a singular example whereby the ability of a horizontally acquired gene to confer a selective advantage upon a recipient organism is dictated by quantitative differences in the biochemical activities of ancestral proteins.

## Results

### Evidence That *pmrD* Is a Horizontally Acquired Gene

Several lines of evidence support the previously suggested hypothesis that *pmrD* is a horizontally acquired gene [25]. First, a search of the completed genomes in the NCBI microbial genomes database for the presence of sequences homologous to the *S. enterica* 14028s or *E. coli* K12 MG1655 PmrD protein (conducted on May 2011) showed that *pmrD* is limited to enteric bacteria of the *Klebsiella*, *Citrobacter*, *Salmonella* and *Escherichia* lineages (Figure S1A). Second, a BLAST search identified a *pmrD*-like gene encoded on plasmid pKP187 from the nitrogen-fixing endophyte *K. pneumoniae* 342 [26], which specifies a product displaying ∼30% amino acid identity to the *Salmonella* and *E. coli* PmrD proteins (Figure S1). The presence of *pmrD* on a mobile genetic element could provide a means for its transfer among bacteria. Third, phylogenetic analysis of *pmrD* homologs present in enteric bacteria revealed that the *pmrD* neighbor-joining tree was similar to the species phylogeny constructed using four housekeeping genes (*gapA*, *groEL*, *gyrA* and *pgi*). The only exception was the *Citrobacter pmrD* gene, which clustered with the homolog identified in *K. pneumoniae* 342 pKP187 (Figure S1A) and this incongruence with the accepted species phylogeny is likely a consequence of horizontal gene transfer. Fourth, the *pmrD* gene is located in the same chromosomal context in the *Citrobacter*, *Salmonella* and *Escherichia* genomes, which is different from that of the *Klebsiella* genome (Figure S1B). Collectively, these analyses suggest that the *pmrD* gene was horizontally acquired by one or several transfer events.

### The *E. coli* PmrD Protein Connects the PhoP/PhoQ and PmrA/PmrB Systems in *Salmonella* But Not in *E. coli*

**Figure 2:**
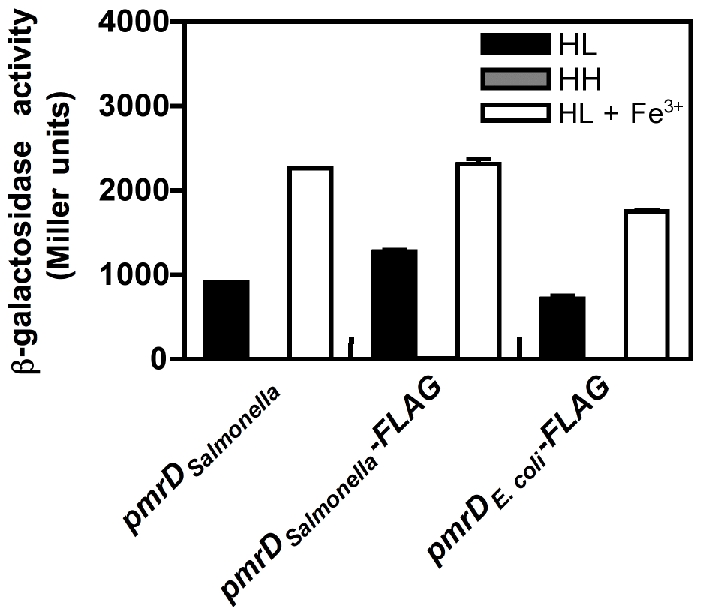
The *E. coli* PmrD protein enables transcription of PmrA-activated genes in *Salmonella* experiencing low Mg^2+^. β-galactosidase activity (Miller units) produced by isogenic *Salmonella* strains harboring a chromosomal *pbgP*-*lac* transcriptional fusion and the wild-type *Salmonella pmrD* gene (EG9241), a 3′ FLAG-tagged *Salmonella pmrD* gene (EG13942), or a 3′ FLAG-tagged *E. coli pmrD* gene (EG13941) transcribed from the normal *Salmonella pmrD* promoter and chromosomal location. Bacteria were grown in N-minimal medium at pH 7.7 with 10 µM Mg^2+^ (HL), 10 mM Mg^2+^ (HH) or 10 µM Mg^2+^ and 100 µM Fe^3+^ (HL+Fe^3+^). Data correspond to the mean of three independent experiments performed in duplicate and error bars show standard deviation.

To explore the possibility that the *E. coli* PmrD protein might activate the PmrA/PmrB system in *Salmonella* experiencing low Mg^2+^ (even though it does not function in this capacity in *E. coli* [23]), we engineered a *Salmonella* strain that expresses a FLAG-tagged *E. coli pmrD* gene under the control of the *Salmonella pmrD* promoter in the normal chromosomal location. The resulting strain transcribed the PmrA-activated *pbgP* gene in low Mg^2+^, like an isogenic strain expressing *Salmonella*'s own *pmrD* gene or a FLAG-tagged version of it (Figure 2). As expected, all three strains expressed *pbgP* to high levels when grown in the presence of Fe^3+^, which activates the PmrA/PmrB system in a PmrD-independent manner [22], but not under non-inducing (high Mg^2+^ and no Fe^3+^) conditions (Figure 2). Moreover, a *Salmonella pmrD* mutant harboring a plasmid encoding the *E. coli pmrD* gene or a FLAG-tagged version of it transcribed *pbgP* in low Mg^2+^, unlike cells harboring the plasmid vector (Figure S2). These results indicate that the ability of the *E. coli* PmrD protein to connect the PhoP/PhoQ and PmrA/PmrB systems is context-dependent: it functions in *Salmonella*, but surprisingly, not in *E. coli*.

### The *E. coli* PmrB Protein Exhibits Higher Phosphatase Activity towards PmrA-P Than the *Salmonella* PmrB Protein

Why does expression of the *E. coli pmrD* gene result in *pbgP* transcription in *Salmonella* but not in *E. coli* during growth in low Mg^2+^? When bacteria experience inducing conditions for the ancestral PmrA/PmrB system, the sensor PmrB promotes the phosphorylated state of the DNA binding protein PmrA [22], [27], resulting in transcription of PmrA-activated genes [28]. In addition to being an autokinase and phosphotransferase, PmrB is a PmrA-P phosphatase, an activity present primarily under non-inducing conditions [29]. The PmrD protein, which is produced in low Mg^2+^, promotes the phosphorylated state of PmrA by antagonizing PmrB-mediated dephosphorylation of PmrA-P [21]. Thus, we hypothesized that the orthologous PmrA and/or PmrB proteins of these two species might differ in one or more of their biochemical properties, resulting in lower levels of PmrA-P – the target of PmrD – in *E. coli* than in *Salmonella*. To evaluate this possibility, we compared the behaviors of the purified cytoplasmic domain of the PmrB protein (PmrB_c_) from the two species because it retains all the known enzymatic activities of the full-length PmrB protein, and because PmrB_c_ expression recapitulates the PmrD-dependent activation of PmrA-regulated genes displayed by the full-length PmrB protein *in vivo* (Figure S3) [21].

First, we examined the autophosphorylation rate of the two proteins and found that the ATP concentration at which it was half maximal was two-fold higher for the *E. coli* PmrB_c_ protein than for the *Salmonella* PmrB_c_ protein (Figure S4A; see Table 1 for *K_M_* values). In addition, the *E. coli* PmrB_c_ protein autophosphorylated at a rate that was two-fold higher than the *Salmonella* PmrB_c_ protein (Figure S4B; see Table 1 for *k_observed_* values). Given that the intracellular ATP concentration is ∼3 mM in *E. coli* [30], which is >30 times the experimentally determined *K_M_* for ATP of the *Salmonella* and *E. coli* PmrB_c_ proteins (Table 1), we anticipate that these PmrB orthologs autophosphorylate at similar rates *in vivo*.

**Table 1:** Kinetic properties of PmrB_c_ autophosphorylation.

Parameter	*E. coli*	*Salmonella*
**Apparent ** ***K_M_*** ** (µM)**	94±9	46±4
***k_observed_*** ** (min-1)**	0.07±0.01	0.03±0.003

**Figure 3:**
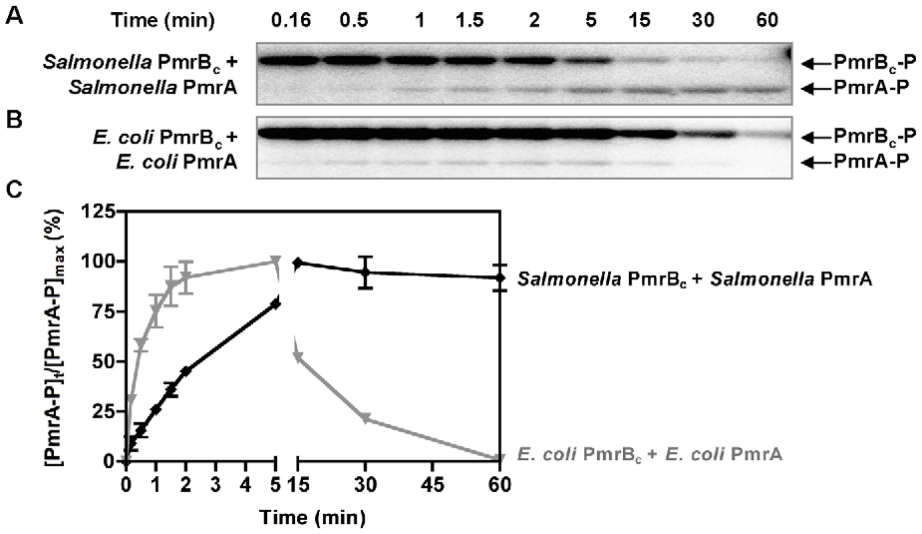
Phosphotransfer profiles reflect differences between the phosphatase activities of the *Salmonella* and *E. coli* PmrB_c_ proteins. (A–B) Levels of PmrB_c_-P and PmrA-P following incubation of *Salmonella* or *E. coli* PmrB_c_-P (5 µM) with *Salmonella* or *E. coli* PmrA (10 µM) proteins at the times indicated at the top of the figure according to the protocols described in Materials and Methods. (C) Quantitation of the phosphotransfer assays shown in (A–B). The plot depicts the level of PmrA-P relative to the maximum achieved as a function of time. Data correspond to the mean values of three independent experiments and error bars show standard deviation.

We next analyzed phosphotransfer from the *Salmonella* PmrB_c_ protein to the *Salmonella* PmrA protein. The amount of PmrA-P increased steadily, reaching a maximum at 30 min (Figure 3A and 3C). The increase in the levels of PmrA-P was accompanied by a decrease in the levels of PmrB_c_-P (Figure 3A). By contrast, when the phosphotransfer reaction was performed with the PmrB_c_-P and PmrA proteins from *E. coli*, there was a rapid increase in the levels of PmrA-P during the first 5 min, followed by a steady decrease (Figure 3B and 3C). The decrease in PmrA-P levels was not due to a contaminating protease because the total PmrA amounts remained constant throughout the course of the reaction (Figure S4C). The different rates of dephosphorylation of the orthologous PmrA-P proteins after reaching their maximal values reflect the source of the PmrB_c_ proteins (as opposed to resulting from differences in the rate of spontaneous hydrolysis of the phosphoryl group from the PmrA-P proteins) because the stabilities of the *Salmonella* and *E. coli* PmrA-P proteins were similar when the PmrB_c_ proteins were omitted from the reactions (Figure S4D). Cumulatively, these experiments suggested that the *Salmonella* and *E. coli* PmrB_c_ proteins differ in their phosphatase activity towards PmrA-P.

We established that the rate with which PmrB_c_ dephosphorylates PmrA-P is 10-fold higher for the reaction with the *E. coli* PmrB_c_ and PmrA-P proteins than that displayed by the *Salmonella* PmrB_c_ and PmrA-P proteins (Figure S4E and Table 2). Furthermore, the different rates of PmrB_c_-promoted dephosphorylation of PmrA-P are largely dependent on the source of PmrB_c_ (rather than on PmrA): the *E. coli* PmrB_c_ dephosphorylated the PmrA-P proteins from *Salmonella* and *E. coli* at rates that were 3–10-fold higher than those with which the *Salmonella* PmrB_c_ protein dephosphorylated PmrA-P from *Salmonella* or from *E. coli* (Figure S4E and Table 2). In sum, the enhanced phosphatase activity of the *E. coli* PmrB_c_ towards PmrA-P (Figure 3 and Table 2) is predicted to generate lower levels of PmrA-P in *E. coli* than in *Salmonella*, which would result in reduced transcription of PmrA-activated genes [31].

**Table 2:** Rate constants for PmrB_c_-mediated dephosphorylation of PmrA-P, *k_observed_* (min^−1^).

	*E. coli* PmrBc	*Salmonella* PmrBc
***E. coli*** ** PmrA**	0.36±0.03	0.03±0.001
***Salmonella*** ** PmrA**	0.14±0.01	0.04±0.004

### Increased Phosphatase Activity in a Chimeric *Salmonella* PmrB Protein Harboring a Subdomain from the *E. coli* PmrB Protein

To explore the molecular basis for the differences in phosphatase activity exhibited by the *Salmonella* and *E. coli* PmrB proteins, we carried out homology modeling of their cytoplasmic domains using the structure of the sensor kinase HK853 from *Thermotoga maritima* [32]. This allowed us to predict the secondary structural features of the PmrB homologs and to identify subdomains that differed between the two proteins (Figure S5). Next, we created chimeric PmrB_c_ proteins whereby particular subdomains of the *Salmonella* PmrB_c_ protein were replaced by amino acid sequences corresponding to those from the *E. coli* PmrB_c_ protein, aiming to preserve the secondary structure of PmrB_c_. We then examined the ability of the chimeric proteins to dephosphorylate *Salmonella* PmrA-P.

**Figure 4:**
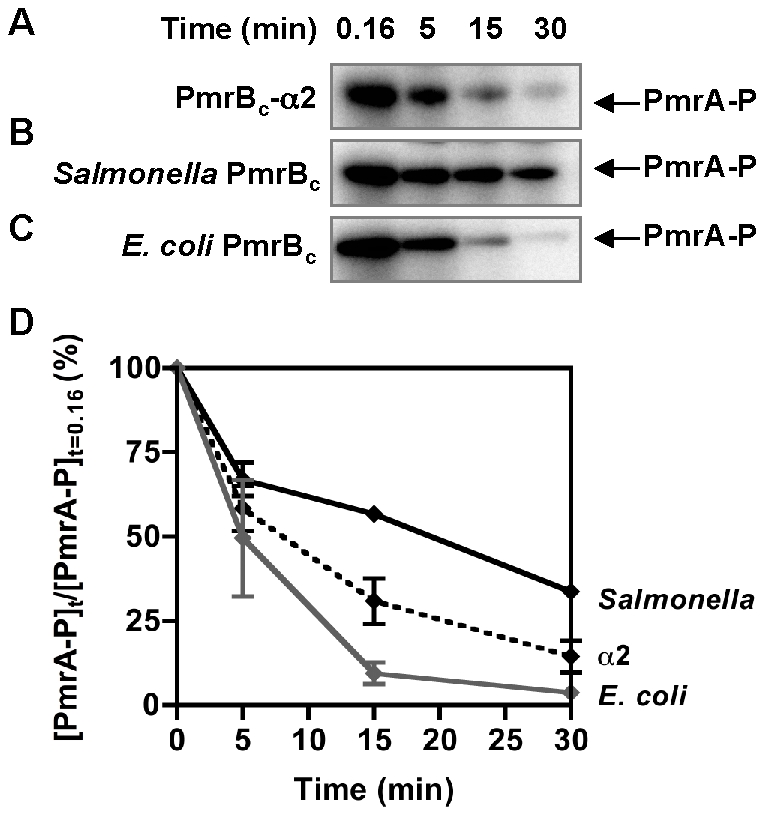
The PmrB_c_-α2 chimera exhibits higher phosphatase activity than the *Salmonella* PmrB_c_ protein. (A–C) Levels of PmrA-P following incubation of *Salmonella* PmrA-P (2.5 µM) with the PmrB_c_-α2 chimera (A), the *Salmonella* PmrB_c_ (B), or the *E. coli* PmrB_c_ (C) (5 µM) proteins for the indicated times. (D) Quantitation of the phosphatase assays shown in (A–C). The graph depicts the level of PmrA-P at the indicated times relative to levels at the start of the reaction. Data correspond to the mean values of three independent experiments and error bars show standard deviation.

The PmrB_c_-α2 chimera, which differs from the wild-type *Salmonella* PmrB_c_ sequence in five residues located in alpha helix 2 (Figure S5), dephosphorylated *Salmonella* PmrA-P at a higher rate than the *Salmonella* PmrB_c_ protein, though not as high as the *E. coli* PmrB_c_ protein (Figure 4). The PmrB_c_-α2 chimera displayed a phosphotransfer profile that was similar to that of the *E. coli* PmrB protein: when phosphorylated PmrB_c_-α2 protein was incubated with *Salmonella* PmrA protein, the levels of PmrA-P increased between 0–5 min and then decreased (Figure S6). By contrast, five other chimeric PmrB_c_ proteins exhibited similar or lower levels of phosphatase activity towards *Salmonella* PmrA-P compared to the *Salmonella* PmrB_c_ protein (Figure S7). As expected, an *E. coli* PmrB_c_-α2 chimeric protein harboring the *Salmonella* PmrB α2 subdomain dephosphorylated *E. coli* PmrA-P at a lower rate than the *E. coli* PmrB_c_ (Figure S8). Our results indicate that residues in alpha helix 2 are critical modulators of PmrB's phosphatase activity.

### An *E. coli* Strain Expressing the *Salmonella pmrB* Gene Transcribes PmrA-Activated Genes and Displays Resistance to Polymyxin B When Grown in Low Mg^2+^

We hypothesized that the heightened phosphatase activity of the *E. coli* PmrB protein may hinder accumulation of high enough levels of PmrA-P, which constitutes the target of the PmrD protein [21], [23], thereby preventing transcription of PmrA-activated genes and resistance to polymyxin B during growth in low Mg^2+^. To test this hypothesis, we engineered isogenic *E. coli* strains expressing either the *Salmonella* or *E. coli pmrB* genes from the normal *E. coli* chromosomal location. We then investigated the PmrD-mediated connectivity between the PhoP/PhoQ and PmrA/PmrB systems by determining the ratio of the mRNA levels corresponding to the PmrA-activated genes *pbgP*, *pmrC* and *pmrG* produced during growth in low Mg^2+^ and those produced following growth in the presence of Fe^3+^ [23], which are PmrD-dependent and -independent processes in *Salmonella*.

**Figure 5:**
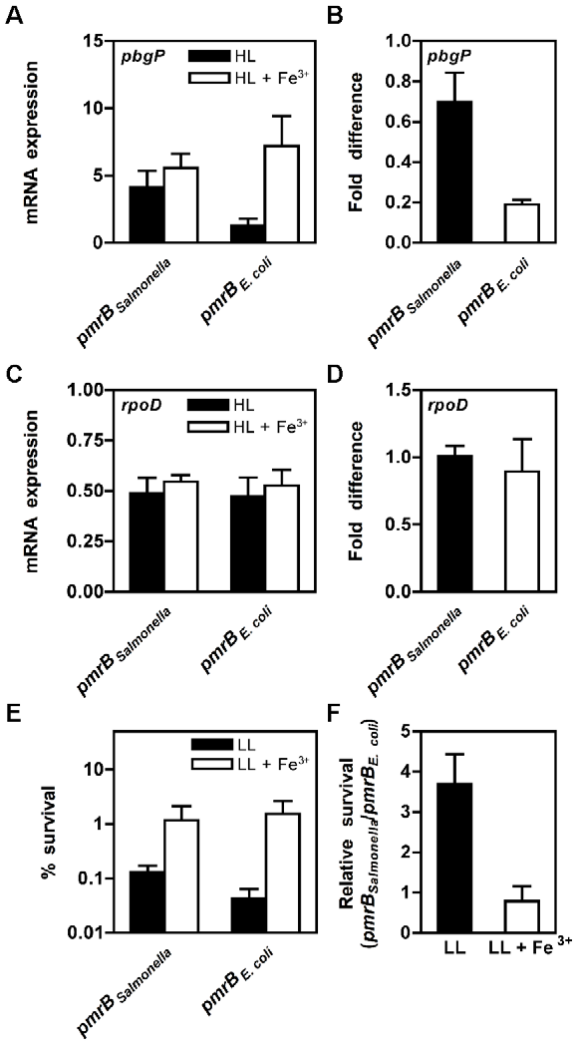
*E. coli* expressing the *Salmonella pmrB* gene display low Mg^2+^-promoted *pbgP* transcription and resistance to polymyxin B. (A–D) The mRNA levels of the PmrA-activated *pbgP* gene (A) or the control *rpoD* gene (C) produced during growth in low Mg^2+^ and those produced following growth in the presence of Fe^3+^ was determined in *E. coli* strains expressing either the *Salmonella pmrB* gene (DC3) or its own *pmrB* gene (DC5). The ratio between the *pbgP* (B) or *rpoD* (D) mRNA levels produced during growth in low Mg^2+^ and those produced following growth in the presence of Fe^3+^ was determined in DC3 or in DC5. RNA samples were prepared from bacteria grown in N-minimal media, pH 7.7 with 10 µM Mg^2+^ (HL) or 10 µM Mg^2+^ and 100 µM Fe^3+^ (HL+Fe^3+^) to OD_600_ 0.5 and the levels of *pbgP* or *rpoD* mRNA were determined by reverse-transcription-qPCR analysis. Data correspond to the mean of at least three independent experiments and error bars represent standard deviation. (E–F) Survival of isogenic *E. coli* strains expressing either the *Salmonella pmrB* gene (DC3) or the *E. coli pmrB* gene (DC5) following incubation in the presence of polymyxin B (2.5 µg/ml). Bacteria were grown in N-minimal medium, pH 5.8, containing 38 mM glycerol with 10 µM Mg^2+^ (LL) or 10 µM Mg^2+^ and 100 µM Fe^3+^ (LL+Fe^3+^). (E) Percentage survival of bacteria following treatment with polymyxin B relative to those incubated in the presence of PBS. (F) Relative survival of *E. coli* strain expressing the *Salmonella pmrB* gene (DC3) to the isogenic *E. coli* strain that expressing *E. coli*'s own *pmrB* gene (DC5). Data correspond to the mean of three independent experiments performed in duplicate and error bars show standard deviation.

PmrD connectivity was higher in the *E. coli* strain expressing the *Salmonella pmrB* gene than in the isogenic strain expressing *E. coli*'s own *pmrB* gene (Figure 5A, 5B; Figure S9A and S9B). An *E. coli* strain harboring the *Salmonella pmrB-α2* gene also exhibited higher PmrD connectivity than the strain expressing the *E. coli pmrB* gene, though not to the level displayed by the strain with the *Salmonella* homolog (Figure S9B). As expected, these strains displayed a similar ratio (∼1) for the PmrA-independent *rpoD* gene (Figure 5C and 5D), which was used as a control. The low PmrD connectivity in the *E. coli* strain harboring its own *pmrB* gene is ascribed to its higher phosphatase activity because an *E. coli* strain expressing the PmrB T156R protein, which dephosphorylates PmrA-P at a lower rate than the *E. coli* PmrB protein *in vitro* (Figure S4F), transcribes PmrA-activated genes in low Mg^2+^ (Figure S9C). The disparate PmrD connectivities were not due to differences in the responses of the orthologous PmrB proteins to Fe^3+^ because these strains displayed similar levels of iron-promoted *pbgP* and *pmrC* transcription (Figure S9D). The heightened expression displayed by the engineered *E. coli* strain harboring the *Salmonella pmrB* gene required the PmrD protein because there was no significant transcription of PmrA-activated genes in a *pmrD* mutant following growth in low Mg^2+^ (Figure S9E).

*E. coli* displays resistance to polymyxin B when grown in the presence of Fe^3+^ (but not following growth in low Mg^2+^) in a process that requires the *pmrA* and *pbgP* genes [23]. We reasoned that the *E. coli* strain expressing the *Salmonella pmrB* gene should display resistance towards polymyxin B when experiencing low Mg^2+^ because PmrA-activated genes are transcribed (Figure 5A). Indeed, the *E. coli* strain expressing the *Salmonella pmrB* gene was ∼4-fold more resistant to polymyxin B than the strain expressing the *E. coli pmrB* gene when bacteria were grown in low Mg^2+^ (Figure 5E and 5F). As expected, both strains displayed similar levels of polymyxin B resistance following growth in the presence of Fe^3+^ (Figure 5E and 5F). Collectively, these findings demonstrate that the source of the *pmrB* gene (*i.e.*, *Salmonella* or *E. coli*) determines the ability of *E. coli* to transcribe PmrA-activated genes and to exhibit polymyxin B resistance in response to low Mg^2+^.

### PmrB Determines the Expression Kinetics of PmrA-Activated Genes

Fe^3+^ activates the PmrA/PmrB system independently of the PmrD protein both in *Salmonella* and *E. coli* [22], [23]. In *Salmonella*, it has been demonstrated that such activation gives rise to a surge in the mRNA levels of PmrA-activated genes whereby the mRNAs increase, peak and then decrease to reach new steady-state levels [27], and that this reflects changes in the amount of PmrA-P protein [27]. Because the *E. coli* PmrB protein displays higher phosphatase activity than the *Salmonella* homolog (Table 2), we wondered whether the levels of PmrA-P, and thus the kinetics of PmrA-dependent gene expression, might differ temporally in *E. coli* when it expresses the *Salmonella* (instead of the *E. coli*) *pmrB* gene.

**Figure 6:**
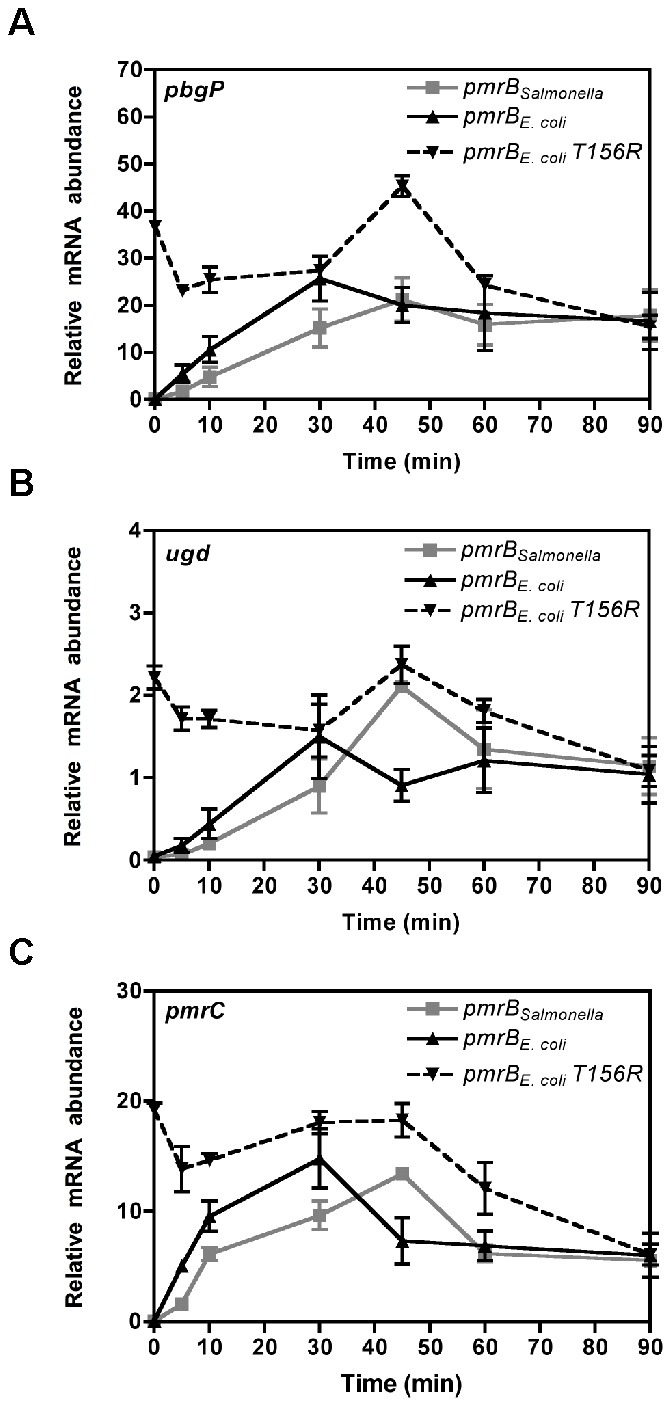
The source of *pmrB* determines activation kinetics of PmrA-controlled genes. The mRNA levels of the PmrA-activated *pbgP* (A), *pmrC* (B) and *ugd* (C) genes from isogenic *E. coli* strains deleted in the *pmrD* gene and expressing either the *Salmonella pmrB* gene (DC9), the *E. coli pmrB* gene (DC11) or the *E. coli pmrB T156R* gene (DC116) were determined by reverse-transcription-qPCR analysis. We used RNA prepared from bacteria grown in medium containing 10 mM Mg^2+^, shifted to medium containing 10 µM Mg^2+^ and 100 µM Fe^3+^ and harvested at the designated times. Expression levels were normalized to those of the 16S ribosomal RNA gene. Data correspond to at least four independent experiments and error bars show standard deviation.

When bacteria were shifted to media containing Fe^3+^, transcription of PmrA-activated genes increased during the first 30 min and then decreased to reach steady-state levels in the *E. coli* strain harboring its own *pmrB* gene (Figure 6). By contrast, in the *E. coli* strain expressing the *Salmonella pmrB* gene, the mRNA levels of PmrA-activated genes reached a peak at 45 min followed by a decrease to attain steady-state levels (Figure 6). To determine whether the distinct temporal transciption profiles of PmrA-activated genes in these strains were due to differences in the rates of PmrB-mediated PmrA-P dephosphorylation, we examined mRNA levels in an *E. coli* strain expressing the *E. coli* PmrB T156R protein, which is compromised in its phosphatase activity (Figure S4F). The mRNA levels of PmrA-activated genes were high even when bacteria were grown high Mg^2+^ and no Fe^3+^, consistent with the notion that PmrB functions primarily as a PmrA-P phosphatase under these conditions (Figure 6) [21]. When bacteria were shifted to media containing Fe^3+^, transcription of PmrA-activated genes increased and peaked at 45 min before decreasing to reach steady-state levels (Figure 6). Altogether, our findings suggest that the higher phosphatase activity displayed by the *E. coli* PmrB protein results in PmrA-dependent transcription being turned off at an earlier timepoint in the *E. coli* strain expressing its own *pmrB* gene than in the isogenic strains harboring the *Salmonella* homolog or the *E. coli pmrB T156R* gene.

## Discussion

### Ancestral Genes Can Dictate the Functionality of Horizontally Acquired Genes

Horizontal gene transfer can endow bacteria with new capabilities, such as the ability to resist killing by an antibiotic or to gain access to a eukaryotic cell, and thus, it can potentially expand their ecological niches [1], [5]–[7]. We have now established that variation in the amino acid sequences of the *Salmonella* and *E. coli* PmrB sensors results in quantitative differences in their biochemical activities (Figure 3 and Table 2) and governs the ability of these bacteria to survive killing by polymyxin B in low Mg^2+^ (Figure 5). We proposed that, by generating different amounts of PmrA-P, the different phosphatase activities of the PmrB orthologs determine the ability of PmrD to activate the PmrA/PmrB system (Figure 2 and Figure 5) [23]. Therefore, unlike self-contained horizontally acquired gene clusters that readily confer a new trait upon introgression [1], [8], [9], the functionality of a horizontally acquired gene product operating on ancestral pathways depends largely upon the biochemical properties of existing ancestral proteins, which can act in a variety of ways [33]–[35].

The effect of genetic context on protein function is not limited to horizontally acquired genes in bacteria. A recent genome-wide survey of genes that were essential in one budding yeast strain but not in another strain demonstrated that this essential character was due to distinct genetic interactions, which differed in a strain-specific manner [36]. In higher eukaryotes, the development of complex traits involves multiple interacting genetic loci; thus, the effect of a certain gene may require other genes controlling the same trait [37]. Taken together, the contribution of a given gene to a particular phenotype is largely determined by its interactions with other genes functioning in the same genetic network.

### Particular Combinations of Gene Interactions Underlie Qualitatively Similar Traits

Given that the heightened phosphatase activity of the *E. coli* PmrB protein prevents the *E. coli* PmrD protein from promoting the accumulation of sufficient levels of PmrA-P (Figure 3 and Figure 5, Table 2), why is it that expression of the *Salmonella pmrD* gene allows transcription of PmrA-activated genes in *E. coli* experiencing low Mg^2+^ [23]? Sequence analysis of the *pmrD* genes suggested that selection and divergence between the *Salmonella* and *E. coli pmrD* loci [23] allowed the *Salmonella* PmrD, but not the *E. coli* ortholog, to connect the PhoP/PhoQ and PmrA/PmrB systems in *E. coli*. These results indicate that particular combinations of *pmrD* and *pmrB* alleles dictate whether bacteria experiencing low Mg^2+^ accumulate sufficient levels of PmrA-P to promote expression of PmrA-dependent genes and to resist killing by polymyxin B (Figure 2 and Figure 5) [23]. Our work, together with studies of gene interactions in model organisms such as budding yeast [36] and analyses of the existing phenotypic variation within natural populations [38], demonstrate that different combinations of particular alleles within a genetic network can lead to qualitatively similar phenotypes in related organisms [37]. Therefore, understanding the phenotypic variation that exists within or between species demands going beyond the study of single-gene effects to investigating how genetic variation at different loci influence one another.

### Quantitative Differences in the Phosphatase Activity of Sensor Proteins Determine Expression Kinetics

Organisms respond to a stress condition by altering their gene expression programs and/or behaviors with temporal dynamics specified by their signaling systems [27], [39], [40]. The distinct dynamics that distinguish the responses of closely related organisms are typically ascribed to differences in the genetic architecture of their signaling systems [41]. For example, the enteric species *Yersinia pestis*, *K. pneumoniae* and *S. enterica* utilize different genetic architectures to promote transcription of the *pbgP* gene when experiencing low Mg^2+^, resulting in distinct expression profiles [25], [42], [43]. We now provide a singular example where variation in the biochemical activity of orthologous signaling proteins can affect expression dynamics in genetic systems with identical architectures. The identified differences in phosphatase activities exhibited by the *Salmonella* and *E. coli* PmrB proteins lead to dissimilar kinetics of PmrA-dependent gene expression (Figure 6, Table 2), which could affect resistance to Fe^3+^ [44] and contribute to the distinct ability of these two species to survive in soil [45].

The *Salmonella* and *E. coli* PmrB proteins are the first example, to our knowledge, where amino acid changes between orthologous bifunctional sensor kinases bring about dramatic differences in phosphatase activity (Table 2) without much effect on autokinase activity (Table 1). These two enzymatic activities are carried out by the cytoplasmic portion of these proteins, which consists of the dimerization and histidine phosphotransfer (DHp) domain joined by a flexible linker to the catalytic and ATP-binding (CA) domain [32], [46], [47]. Amino acid residues identified in the DHp [48]–[51] and CA domains [52] of other sensor kinases as being important for phosphatase activity are conserved between the *Salmonella* and *E. coli* PmrB proteins. This was expected because mutation of these residues usually abolishes phosphatase activity and both the *Salmonella* and *E. coli* PmrB proteins exhibit phosphatase activity towards PmrA-P, albeit to different levels (Table 2). One or all the five amino acids residues differing between the orthologous PmrB proteins in the alpha-2 helix (Figure S5) modulate phosphatase activity (Figure 4 and Figure S8). Some of these amino acids lie within a weakly conserved region (termed X-region) of the DHp domain and linker region that is proposed to stimulate sensor protein phosphatase activity by properly orienting the DHp and CA domains towards each other (Figure S5) [49], [51]. Thus, one or more of these five amino acids might also modulate contacts between the DHp and CA domains of the PmrB proteins.

### Evolution of an Ancestral Regulatory System Targeted by a Horizontally Acquired Gene Product

We have established that quantitative differences between the biochemical properties of the orthologous PmrB proteins control the functionality of the *pmrD* gene product in present-day *Salmonella* and *E. coli*. Yet, when did these genetic events (*i.e.* horizontal acquisition of *pmrD* and mutational changes in *pmrB*) occur in the evolutionary history of enteric species? And how might the integration of horizontally acquired genes into ancestral regulatory circuits drive their evolution and facilitate bacterial adaptation to particular environments?

The *pmrD* gene appears to have been acquired by the common ancestor to the enteric species *K. pneumoniae*, *Citrobacter spp.*, *S. enterica* and *E. coli* and incorporated into the regulatory circuit of the ancestral PhoP/PhoQ system because it is present in these four species but absent from all other examined enteric bacteria (Figure S1) [25]. This ancestral organism likely transcribed PmrA-activated genes in low Mg^2+^ via the PmrD-mediated pathway as this ability is retained in *Klebsiella* and *Salmonella* [12], [25]. After the *Salmonella* and *E. coli* lineages diverged, the ability to make low Mg^2+^-dependent, PmrA-controlled modifications of the LPS was retained by *Salmonella* but lost in *E. coli*, perhaps contributing towards their survival and proliferation in distinct host and non-host environments [45]. This difference can be attributed, in part, to selection at the *pmrD* locus and divergence of the *Salmonella* and *E. coli* PmrD orthologs because expression of the *Salmonella pmrD* gene allows transcription of PmrA-activated genes under low Mg^2+^-inducing conditions in *E. coli* [23]. We further propose that selection for a *Salmonella* PmrB that has low phosphatase activity may have served to maintain the PmrD-mediated connection between the PhoP/PhoQ and PmrA/PmrB systems (Table 2), thus enabling *Salmonella* to display the LPS modifications, including those resulting in polymyxin B resistance in environments denoted by low Mg^2+^ [12], [21]. By contrast, the accumulation of PmrA-P in low Mg^2+^ may be detrimental to *E. coli*'s lifestyle because a mutant that constitutively activates the PmrA/PmrB system exhibits increased susceptibility to the bile detergent deoxycholic acid [53], which *E. coli* may experience in the mammalian intestine [54]. This may have driven selection for a PmrB protein with a higher phosphatase activity in *E. coli* (Table 2). Indeed, molecular analysis of the *pmrB* gene from natural isolates corresponding to the *Salmonella* reference collection C (SARC) [55] and natural isolates from the *E. coli* standard reference collection (ECOR) [56] revealed that the *pmrB* gene is evolving in a non-neutral fashion (Table S1).

In *Salmonella*, further integration of the *pmrD* gene into the regulatory circuits of the ancestral PhoP/PhoQ and PmrA/PmrB systems has entailed the evolution of a negative feedback loop where PmrA-P represses PhoP-promoted *pmrD* transcription, thus preventing excessive expression of PmrA-activated genes (Figure 1A) [29]. This feedback loop is absent in *E. coli* because the PmrD protein does not activate the PmrA/PmrB system in this species following growth in low Mg^2+^ (Figure 1B) [23]. Our work, and that of others [57], suggests that changes in ancestral regulatory and metabolic networks arising from horizontal gene transfer contribute to the survival of bacterial species in their particular niches.

### Concluding Remarks

In sum, genetic changes are often contingent upon those previously fixed within an evolutionary lineage [58]. For example, evolution of the ability to metabolize citrate in an experimental population of *E. coli* was shown to require the occurrence of earlier mutations, which potentiated the appearance of the latter mutation giving rise to the novel trait [59]. Our findings now suggest that horizontal acquisition of the *pmrD* gene might have potentiated selection at the *Salmonella* and *E. coli pmrB* loci, further cementing the ability of *Salmonella*, but not *E. coli*, to express PmrA-activated genes and to resist killing by polymyxin B in the presence of low Mg^2+^.

Finally, the behavior of an organism, or its survival in particular ecological niches, is often deduced from the presence or absence of genes and/or the predicted functions of the encoded gene products, which are based on experiments typically carried out with model organisms. However, more complex mechanisms could link an organism's genotype to its phenotype [60]. We have now established that quantitative differences in the biochemical activities of an ancestral protein can determine whether a horizontally acquired gene product connects different regulatory systems, thereby affecting the ability of a bacterial species to survive killing by cationic antimicrobial peptides. Consequently, subtle quantitative differences in the biochemical activities of conserved orthologous proteins, which are not easily predicted by sequence comparison and identification of conserved catalytic residues, can give rise to phenotypic variation between and within closely related species [38], [61]. Our work demonstrates that protein function can only be appreciated in the context of other proteins operating within the same genetic network, and underscores the need to experimentally verify predictions derived from comparative genomics.

## Materials and Methods

### Bacterial Strains, Plasmids, and Growth Conditions

*S. enterica* serovar Typhimurium strains were derived from the wild-type strain 14028s and *E. coli* strains from the wild-type strain MG1655. Bacteria were grown at 37°C with aeration in Luria-Bertani (LB) broth or in N-minimal media (pH 7.7), supplemented with 0.1% casamino acids, 38 mM glycerol, 10 µM or 10 mM MgCl_2_ and 100 µM FeSO_4_ [62]. When necessary, antibiotics were added at the following final concentrations: ampicillin, 50 µg/ml; chloramphenicol, 20 µg/ml; kanamycin, 50 µg/ml, and tetracycline, 10 µg/ml. P1 transduction of *E. coli* strains was performed as described [63]. P22 transduction of *Salmonella* strains was performed as described [64]. *E. coli* DH5α was used as a host for the preparation of plasmid DNA. We describe the construction of bacterial strains and plasmids as well as other molecular biology procedures in the Text S1. Bacterial strains and plasmids used in this study are listed in Table S2 and S3, respectively.

### β-Galactosidase Assay

β-galactosidase assays were carried out in triplicate, and the activity was determined as described [63]. Data correspond to mean values of three independent experiments performed in duplicate.

### Autokinase, Phosphotransferase, and Phosphatase Assays

Biochemical assays were carried out as in [21] and a detailed description is provided in Text S1.

### RNA Isolation and Real-Time PCR to Determine Transcript Levels

*E. coli* cells from overnight cultures grown in N-minimal medium at pH 7.7 with 10 mM MgCl_2_ were washed twice with N-minimal medium containing no Mg^2+^ and added into the appropriate fresh media with 1∶50 dilution. The bacterial cultures were grown to OD_600_ 0.5 in a shaking water bath at 37°C. 0.5 ml aliquots of cells were removed, mixed with RNAprotect Bacteria Reagent (Qiagen) for stabilization of RNA, and total RNA was isolated using RNeasy Kit (Qiagen) with on-column DNase treatment. cDNA was synthesized using TaqMan (Applied Biosystems) and random hexamers. Quantification of transcripts was carried out by real-time PCR using SYBR Green PCR Master Mix (Applied Biosystems) in an ABI 7500 Sequence Detection System (Applied Biosystems). The relative amount of cDNA was determined using a standard curve obtained from PCR with serially diluted genomic DNA, and results were normalized to the levels of 16S ribosomal RNA. Data shown is an average from at least three independent experiments.

### Polymyxin B Susceptibility Assay

Polymyxin B susceptibility assays were carried out as described in [23]. Data correspond to mean values of three independent experiments performed in duplicate.

### Homology Searches and Phylogenetic Analyses of the *pmrD* Gene

To identify homologs of the *pmrD* gene, the deduced PmrD protein sequences from *E. coli* K12 MG1655 and *S. enterica* serovar Typhimurium 14028s were used as query to search the NCBI microbial genomes database as well as the NCBI non-redundant nucleotide database using TBLASTN. In parallel, the UniProt database was searched for homologs using BLASTP [65]. Similar results were obtained in these searches and the protein sequences of the homologs were used for phylogenetic analyses. The sequences of the *pmrD* gene were aligned using ClustalX2 [66] and phylogenetic trees were constructed using the neighbor-joining algorithm with default parameters and 1000 bootstrap replicates using MEGA 4 [67]. For comparison, the sequences of 4 housekeeping genes (*gapA*, *groEL*, *gyrA* and *pgi*) [68] were concatenated, aligned using ClustalX2 [66] and used for construction of a species tree.

### Accession Numbers

GenBank accession numbers are available in Table S4.

## Supporting Information

Figure S1 Phylogeny of the *pmrD* gene and its genetic context in enteric bacteria. (A) Neighbor-joining tree of the *pmrD* genes from *Klebsiella*, *Citrobacter*, *Salmonella* and *Escherichia*. For comparison, the sequences of four housekeeping loci were concatenated and similarly used for construction of a neighbor-joining tree. Bootstrap support is indicated above each node, with only values >50% being shown. The tree is drawn to scale, with branch lengths in the same units as those of the evolutionary distances used to infer the phylogenetic tree. (B) Genomic context of the *pmrD* gene in representative *Klebsiella*, *Citrobacter*, *Salmonella* and *Escherichia* strains.
(TIF)

Figure S2 The *E. coli* PmrD protein enables transcription of the PmrA-activated *pbgP* gene in *Salmonella* experiencing low Mg^2+^. (A) Transcript levels of the PmrA-activated *pbgP* gene were determined in a *Salmonella pmrD* strain (EG11491) harboring plasmids pUH-*pmrD_E. coli_-FLAG*, pUH-*pmrD_E. coli_* or the vector pUHE21-2*lacI^q^*. (B) The ratio between the *pbgP* mRNA levels produced during growth in low Mg^2+^ and those produced following growth in the presence of Fe^3+^ was determined in a *Salmonella pmrD* strain (EG11491) harboring plasmids pUH-*pmrD_E. coli_-FLAG*, pUH-*pmrD_E. coli_* or the vector pUHE21-2*lacI^q^*. Bacteria were grown with 0.5 mM IPTG in N-minimal medium at pH 7.7 containing 10 µM Mg^2+^ (HL) or 10 µM Mg^2+^ and 100 µM Fe^3+^ (HL+Fe^3+^). Data correspond to the mean of three independent experiments and error bars show standard deviation.
(TIF)

Figure S3 The *E. coli* PmrB cytoplasmic domain (PmrB_c_) is sufficient for PmrD-dependent transcription of PmrA-activated genes. β-galactosidase activity (Miller units) from a chromosomal *pbgP*-*lac* transcriptional fusion were determined in Δ*pmrB* (EG10065) and Δ*pmrB* Δ*pmrD* (EG12060) *Salmonella* strains harboring pUH-*pmrB*, pUH-*pmrB_c_* or pUHE21-2*lacI^q^*. Bacteria were grown in N-minimal medium at pH 7.7, 0.05 mM IPTG and 50 µg/ml ampicillin with 10 µM Mg^2+^ (HL) or 10 mM Mg^2+^ (HH). Data correspond to the mean of three independent experiments performed in duplicate and error bars show standard deviation.
(TIF)

Figure S4 The total levels of PmrB_c_ and PmrA protein remain constant throughout the course of the phosphotransfer reaction and the PmrA-P proteins display similar stabilities in the absence of PmrB_c_. (A) Levels of *Salmonella* or *E. coli* PmrB_c_-P following incubation of PmrB_c_ (2.5 µM) with ATP at the concentrations indicated at the top of the figure according to the protocols described in Text S1. (B) Levels of *Salmonella* or *E. coli* PmrB_c_-P following incubation of PmrB_c_ (2.5 µM) with 0.75 mM ATP at the times indicated at the top of the figure according to the protocols described in Text S1. (C) Levels of the PmrB_c_ and PmrA proteins from *Salmonella* and *E. coli* were determined by SDS-PAGE and Coomassie staining following phosphotransfer from PmrB_c_-P (5 µM) to PmrA (10 µM) as described in Text S1 using aliquots taken at the indicated times. (D) Levels of PmrA-P proteins from *Salmonella* and *E. coli* were determined as described in Text S1 with aliquots taken at the indicated time points. (E) Levels of PmrA-P following incubation of *Salmonella* or *E. coli* PmrB_c_ (5 µM) with the *Salmonella* or *E. coli* PmrA-P (2.5 µM) proteins for the indicated times according to the protocols described in Text S1. (F) Levels of PmrA-P following incubation of *E. coli* PmrB_c_ T156R (5 µM) with the *E. coli* PmrA-P (2.5 µM) protein for the indicated times according to the protocols described in Text S1.
(TIF)

Figure S5 Primary sequence alignment of the *Salmonella* and *E. coli* PmrB sensor proteins. Residues that differ between the two proteins are highlighted in green (conserved changes) or red (nonconserved changes). A blue vertical dotted line is present before the first amino acid of the PmrB_c_ cytoplasmic domain. The structures of the cytoplasmic domains of the PmrB proteins were predicted using a homology-modeling program (Phyre) [69] and were based on HK853 sensor histidine kinase cytoplasmic domain from *Thermotoga maritima* [32]. The dimerization and histidine phosphotransfer (DHp) domain consists of amino acids 128–200, while the catalytic and ATP-binding (CA) domain consists of amino acids 215–356 and 215–363 from the *Salmonella* and *E. coli* PmrB proteins, respectively. β sheets and α helices in the *Salmonella* and *E. coli* PmrB_c_ proteins that contain non-identical amino acid residues are highlighted by colored boxes, and those that are identical between these proteins are highlighted by grey boxes. The X-region (amino acids 179–202), which is underlined with a black dashed line, was predicted based on sequence alignment to the sensor kinase EnvZ [49], [51].
(TIF)

Figure S6 Phosphotransfer from the PmrB_c_-α2 chimera to the *Salmonella* PmrA protein is similar to that displayed by the *E. coli* PmrB_c_ protein. (A–C) Levels of PmrB_c_-P and PmrA-P following incubation of PmrB_c_-P (5 µM) and PmrA (10 µM) as described in Text S1 with aliquots taken at the times indicated at the top of the figure. Three different reactions were set up with the PmrB_c_-α2 and *Salmonella* PmrA proteins (A), *Salmonella* PmrB_c_ and *Salmonella* PmrA proteins (B), and *E. coli* PmrB_c_ and *Salmonella* PmrA proteins (C). (D) Quantitation of the phosphotransfer assays shown in (A–C). The plot depicts the level of PmrA-P relative to the maximum achieved as a function of time.
(TIF)

Figure S7 The PmrB_c_ chimeras exhibit similar or lower levels of phosphatase activity than the *Salmonella* PmrB_c_ protein. (A–E) The graphs depict the levels of PmrA-P at the indicated times relative to levels at the start of the reaction (t = 0.16 min) following incubation of *Salmonella* PmrA-P (2.5 µM) with the PmrB_c_-α1 protein (A) or PmrB_c_-α3 protein (B) or the PmrB_c_-β2 protein (C) or the PmrB_c_-β4 protein (D) or the PmrB_c_-7aa protein (E). The abilities of the PmrB_c_ chimeric proteins (5 µM) to dephosphorylate PmrA-P were compared to those of the *Salmonella* or *E. coli* PmrB_c_ proteins. Data correspond to the mean values of at least three independent experiments and error bars show standard deviation.
(TIF)

Figure S8 The *E. coli* PmrB_c_–α2 chimera exhibits a lower level of phosphatase activity than the *E. coli* PmrB_c_ protein. (A–C) Levels of PmrA-P following incubation of *E. coli* PmrA-P (2.5 µM) with the *E. coli* PmrB_c_-α2 chimera (A), the *Salmonella* PmrB_c_ (B) or the *E. coli* PmrB_c_ (C) (5 µM) proteins for the indicated times. (D) Quantitation of the phosphatase assays shown in (A–C). The graph depicts the levels of PmrA-P at the indicated times relative to levels at the start of the reaction (t = 0.16 min). Data correspond to the mean values of three independent experiments and error bars show standard deviation.
(TIF)

Figure S9 The *Salmonella pmrB* gene restores low Mg^2+^-promoted transcription of PmrA-activated genes in *E. coli*. (A–C) The ratio between the mRNA levels of the PmrA-activated genes *pmrG*, *pmrC* and *pbgP* produced during growth in low Mg^2+^ and those produced following growth in the presence of Fe^3+^ was determined in *E. coli* expressing the *Salmonella pmrB* gene (DC3) or its own *pmrB* gene (DC5) or the *Salmonella pmrB-*α*2* gene (DC68) or the *E. coli pmrB T156R* gene (DC116) from the normal chromosomal location. RNA samples were prepared from bacteria grown in N-minimal media, pH 7.7 with 10 µM Mg^2+^ (HL) or 10 µM Mg^2+^ and 100 µM Fe^3+^ (HL+Fe^3+^) to OD_600_ of 0.5, and the levels of *pmrG*, *pmrC* or *pbgP* mRNA were determined by reverse-transcription-qPCR analysis. (D) Transcript levels of the PmrA-activated genes *pbgP* and *pmrC* were determined in *E. coli* strains expressing either the *Salmonella pmrB* gene (DC3) or the *E. coli pmrB* gene (DC5). Bacteria were grown in N-minimal media, pH 7.7 with 1 mM Mg^2+^ (HH) or 1 mM Mg^2+^ and 100 µM Fe^3+^ (HH+Fe^3+^) to an OD_600_ 0.5 before RNA samples were prepared for reverse-transcription-qPCR analysis. (E) Transcript levels of the PmrA-activated genes *pbgP* and *pmrC* were determined in *E. coli* strains deleted for the *pmrD* gene and expressing either the *Salmonella pmrB* gene (DC9) or the *E. coli pmrB* gene (DC11). Bacteria were grown in N-minimal media, pH 7.7 with 10 µM Mg^2+^ (HL) or 10 µM Mg^2+^ and 100 µM Fe^3+^ (HL+Fe^3+^) to an OD_600_ 0.5 before RNA samples were prepared for reverse-transcription-qPCR analysis. Data correspond to the mean of at least three independent experiments and error bars represent standard deviation.
(TIF)

Table S1 McDonald-Kreitman test for *pmrB* evolution.
(DOC)

Table S2 Bacterial strains and plasmids used in this study.
(DOC)

Table S3 DNA sequences of primers used in this study.
(DOC)

Table S4 GenBank accession numbers for the *pmrB* genes from *Salmonella* and *E. coli* natural isolates.
(DOC)

Text S1 Supplementary materials and methods.
(DOC)
