# Supplementary figures and images for: Ancestral Genes Can Control the Ability of Horizontally Acquired Loci to Confer New Traits
Source: PLoS Genet. 2011 Jul 21;7(7):e1002184. doi: 10.1371/journal.pgen.1002184 (PMC3140997; doi:10.1371/journal.pgen.1002184)

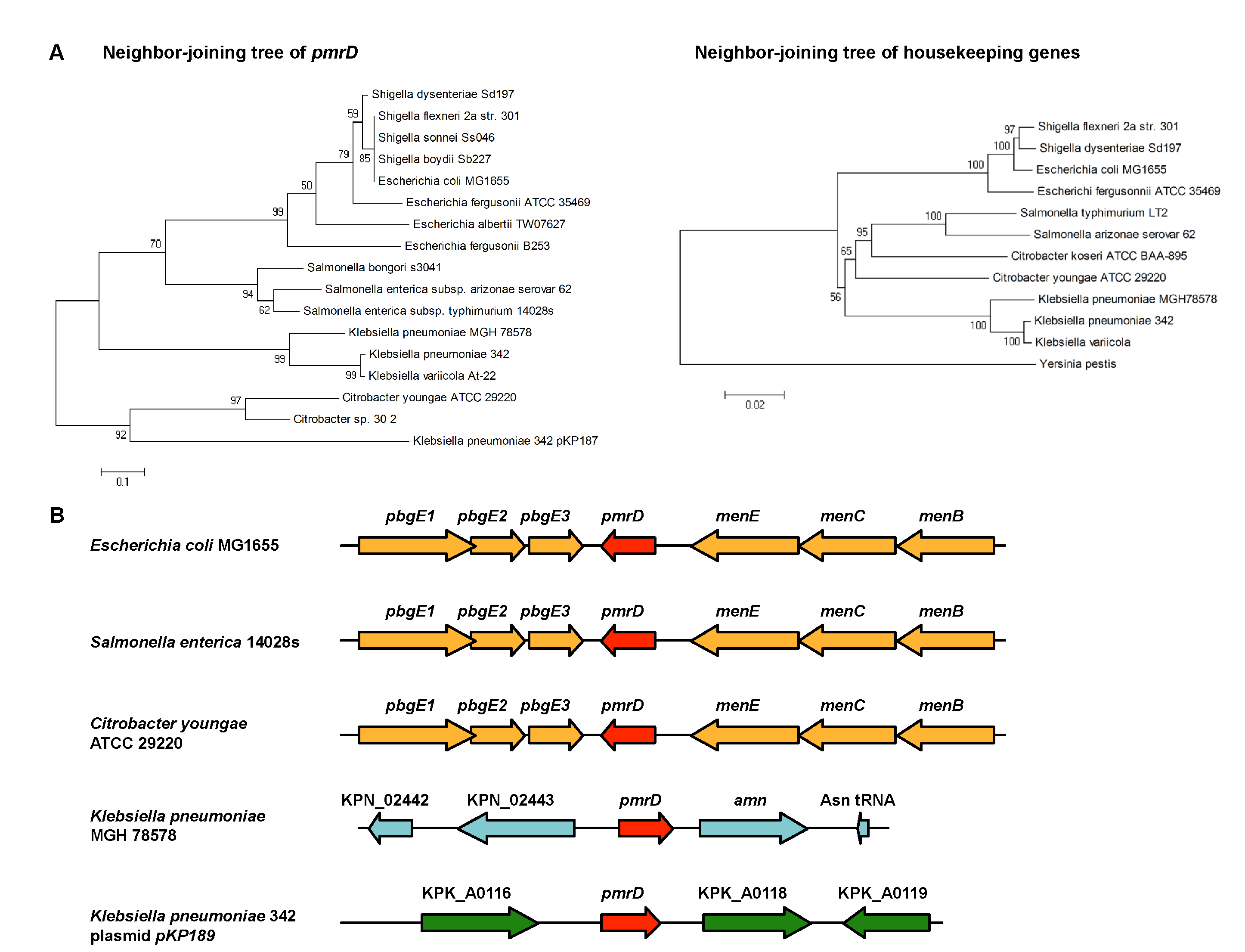

Supplement: Figure S1 — Phylogeny of the pmrD gene and its genetic context in enteric bacteria. (A) Neighbor-joining tree of the pmrD genes from Klebsiella, Citrobacter, Salmonella and Escherichia. For comparison, the sequences of four housekeeping loci were concatenated and similarly used for construction of a neighbor-joining tree. Bootstrap support is indicated above each node, with only values >50% being shown. The tree is drawn to scale, with branch lengths in the same units as those of the evolutionary distances used to infer the phylogenetic tree. (B) Genomic context of the pmrD gene in representative Klebsiella, Citrobacter, Salmonella and Escherichia strains. (TIF) [file pgen.1002184.s001.tif]

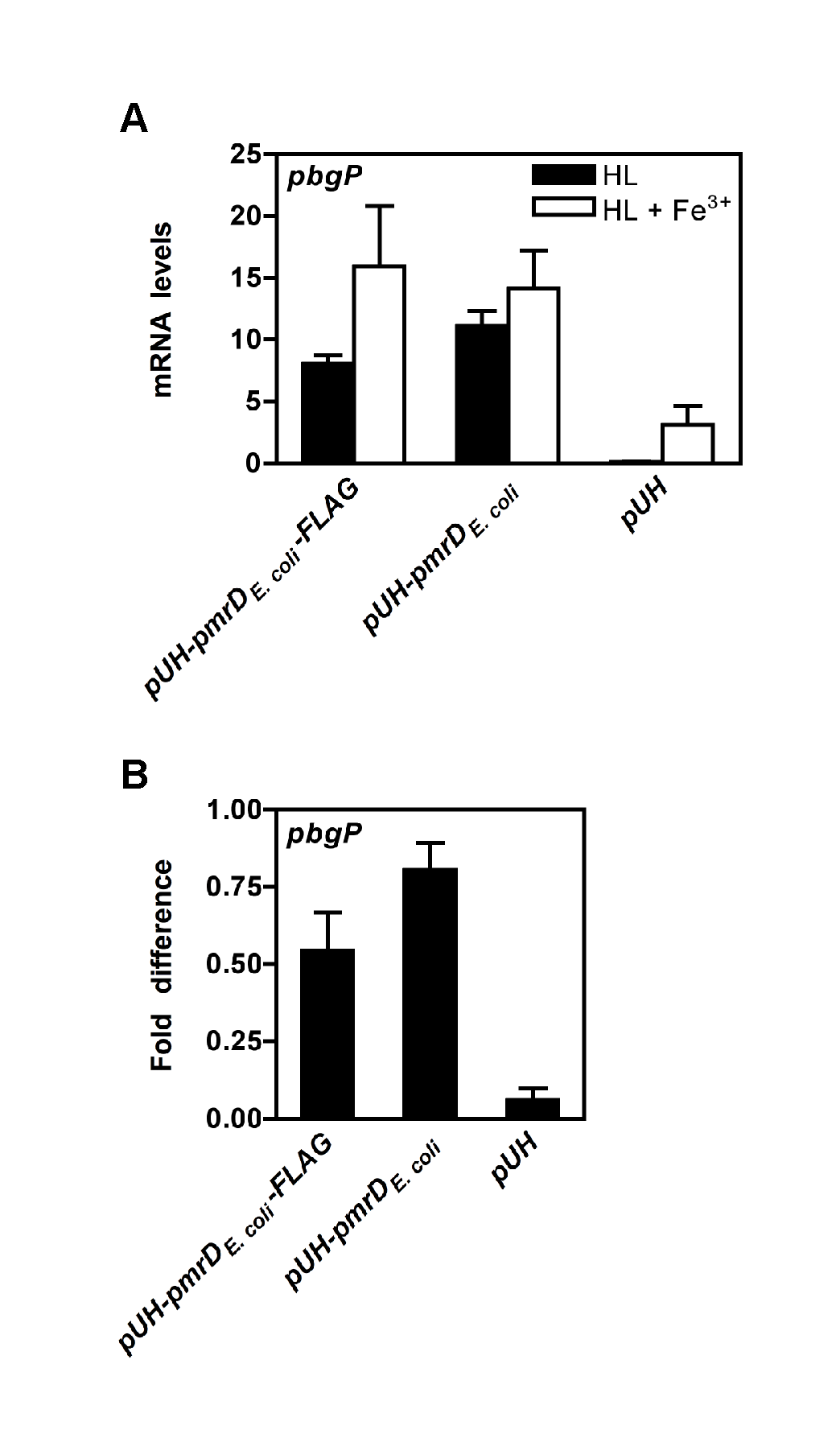

Supplement: Figure S2 — The E. coli PmrD protein enables transcription of the PmrA-activated pbgP gene in Salmonella experiencing low Mg2+. (A) Transcript levels of the PmrA-activated pbgP gene were determined in a Salmonella pmrD strain (EG11491) harboring plasmids pUH-pmrDE. coli-FLAG, pUH-pmrDE. coli or the vector pUHE21-2lacIq. (B) The ratio between the pbgP mRNA levels produced during growth in low Mg2+ and those produced following growth in the presence of Fe3+ was determined in a Salmonella pmrD strain (EG11491) harboring plasmids pUH-pmrDE. coli-FLAG, pUH-pmrDE. coli or the vector pUHE21-2lacIq. Bacteria were grown with 0.5 mM IPTG in N-minimal medium at pH 7.7 containing 10 µM Mg2+ (HL) or 10 µM Mg2+ and 100 µM Fe3+ (HL+Fe3+). Data correspond to the mean of three independent experiments and error bars show standard deviation. (TIF) [file pgen.1002184.s002.tif]

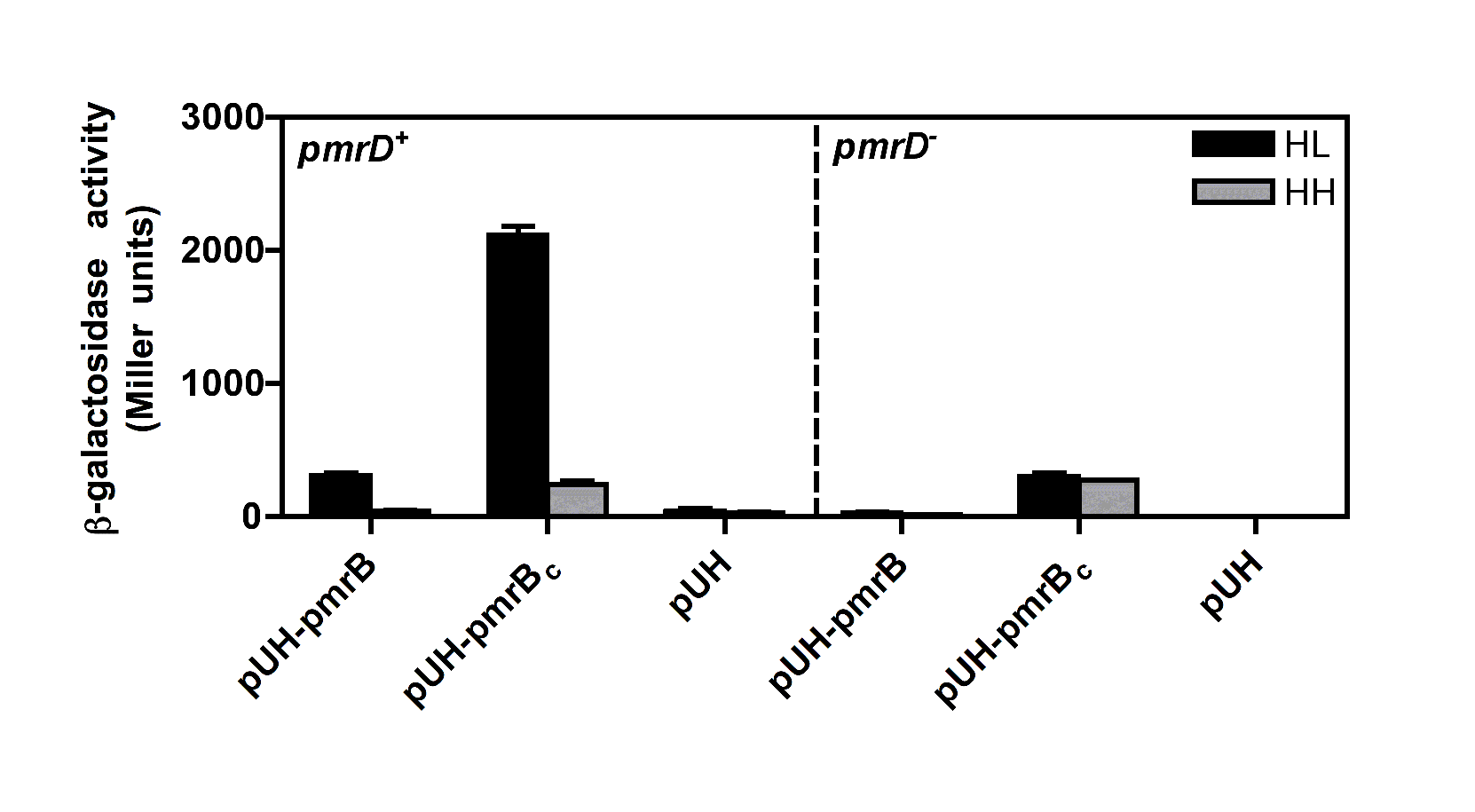

Supplement: Figure S3 — The E. coli PmrB cytoplasmic domain (PmrBc) is sufficient for PmrD-dependent transcription of PmrA-activated genes. β-galactosidase activity (Miller units) from a chromosomal pbgP-lac transcriptional fusion were determined in ΔpmrB (EG10065) and ΔpmrB ΔpmrD (EG12060) Salmonella strains harboring pUH-pmrB, pUH-pmrBc or pUHE21-2lacIq. Bacteria were grown in N-minimal medium at pH 7.7, 0.05 mM IPTG and 50 µg/ml ampicillin with 10 µM Mg2+ (HL) or 10 mM Mg2+ (HH). Data correspond to the mean of three independent experiments performed in duplicate and error bars show standard deviation. (TIF) [file pgen.1002184.s003.tif]

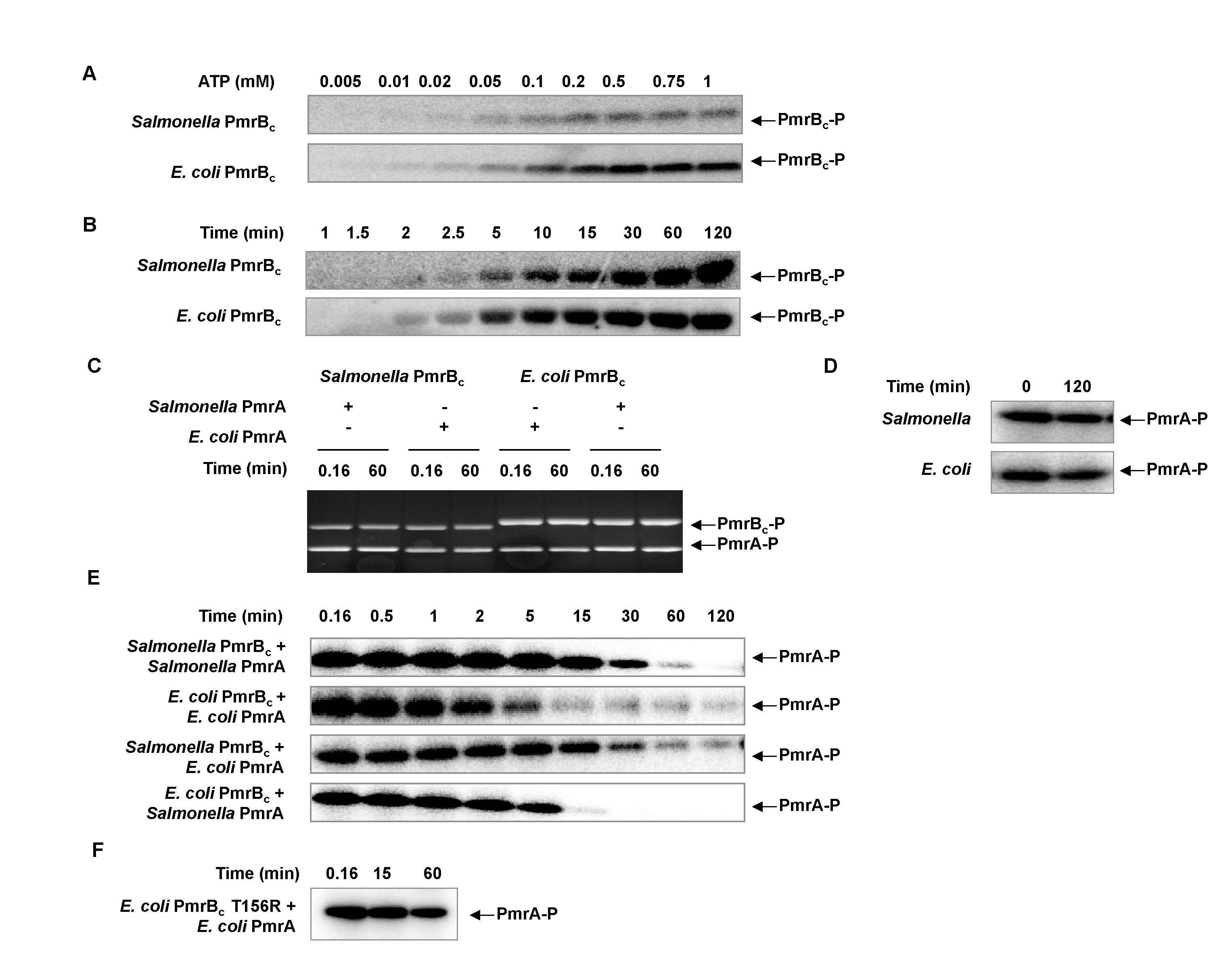

Supplement: Figure S4 — The total levels of PmrBc and PmrA protein remain constant throughout the course of the phosphotransfer reaction and the PmrA-P proteins display similar stabilities in the absence of PmrBc. (A) Levels of Salmonella or E. coli PmrBc-P following incubation of PmrBc (2.5 µM) with ATP at the concentrations indicated at the top of the figure according to the protocols described in Text S1. (B) Levels of Salmonella or E. coli PmrBc-P following incubation of PmrBc (2.5 µM) with 0.75 mM ATP at the times indicated at the top of the figure according to the protocols described in Text S1. (C) Levels of the PmrBc and PmrA proteins from Salmonella and E. coli were determined by SDS-PAGE and Coomassie staining following phosphotransfer from PmrBc-P (5 µM) to PmrA (10 µM) as described in Text S1 using aliquots taken at the indicated times. (D) Levels of PmrA-P proteins from Salmonella and E. coli were determined as described in Text S1 with aliquots taken at the indicated time points. (E) Levels of PmrA-P following incubation of Salmonella or E. coli PmrBc (5 µM) with the Salmonella or E. coli PmrA-P (2.5 µM) proteins for the indicated times according to the protocols described in Text S1. (F) Levels of PmrA-P following incubation of E. coli PmrBc T156R (5 µM) with the E. coli PmrA-P (2.5 µM) protein for the indicated times according to the protocols described in Text S1. (TIF) [file pgen.1002184.s004.tif]

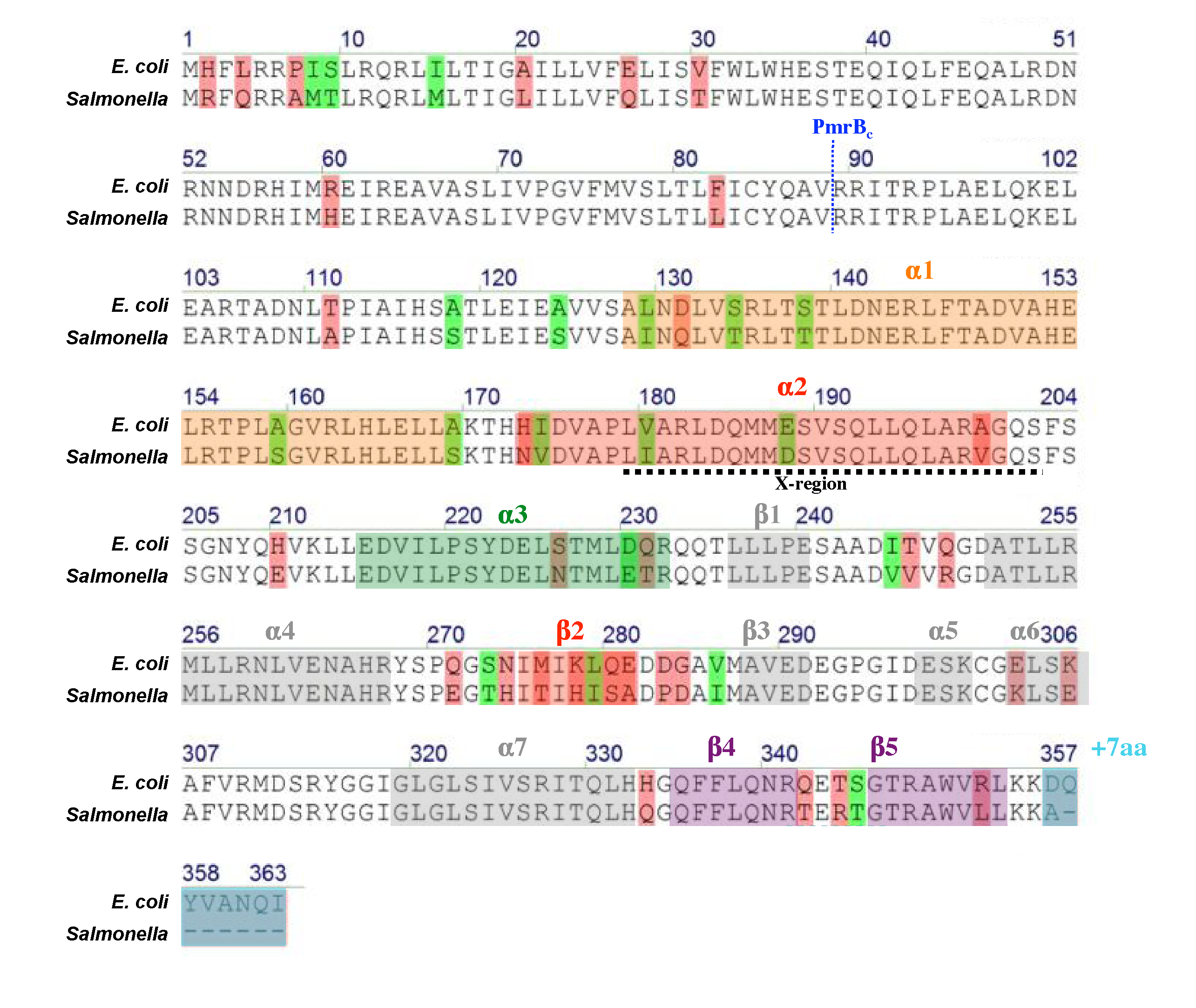

Supplement: Figure S5 — Primary sequence alignment of the Salmonella and E. coli PmrB sensor proteins. Residues that differ between the two proteins are highlighted in green (conserved changes) or red (nonconserved changes). A blue vertical dotted line is present before the first amino acid of the PmrBc cytoplasmic domain. The structures of the cytoplasmic domains of the PmrB proteins were predicted using a homology-modeling program (Phyre) [69] and were based on HK853 sensor histidine kinase cytoplasmic domain from Thermotoga maritima [32]. The dimerization and histidine phosphotransfer (DHp) domain consists of amino acids 128–200, while the catalytic and ATP-binding (CA) domain consists of amino acids 215–356 and 215–363 from the Salmonella and E. coli PmrB proteins, respectively. β sheets and α helices in the Salmonella and E. coli PmrBc proteins that contain non-identical amino acid residues are highlighted by colored boxes, and those that are identical between these proteins are highlighted by grey boxes. The X-region (amino acids 179–202), which is underlined with a black dashed line, was predicted based on sequence alignment to the sensor kinase EnvZ [49], [51]. (TIF) [file pgen.1002184.s005.tif]

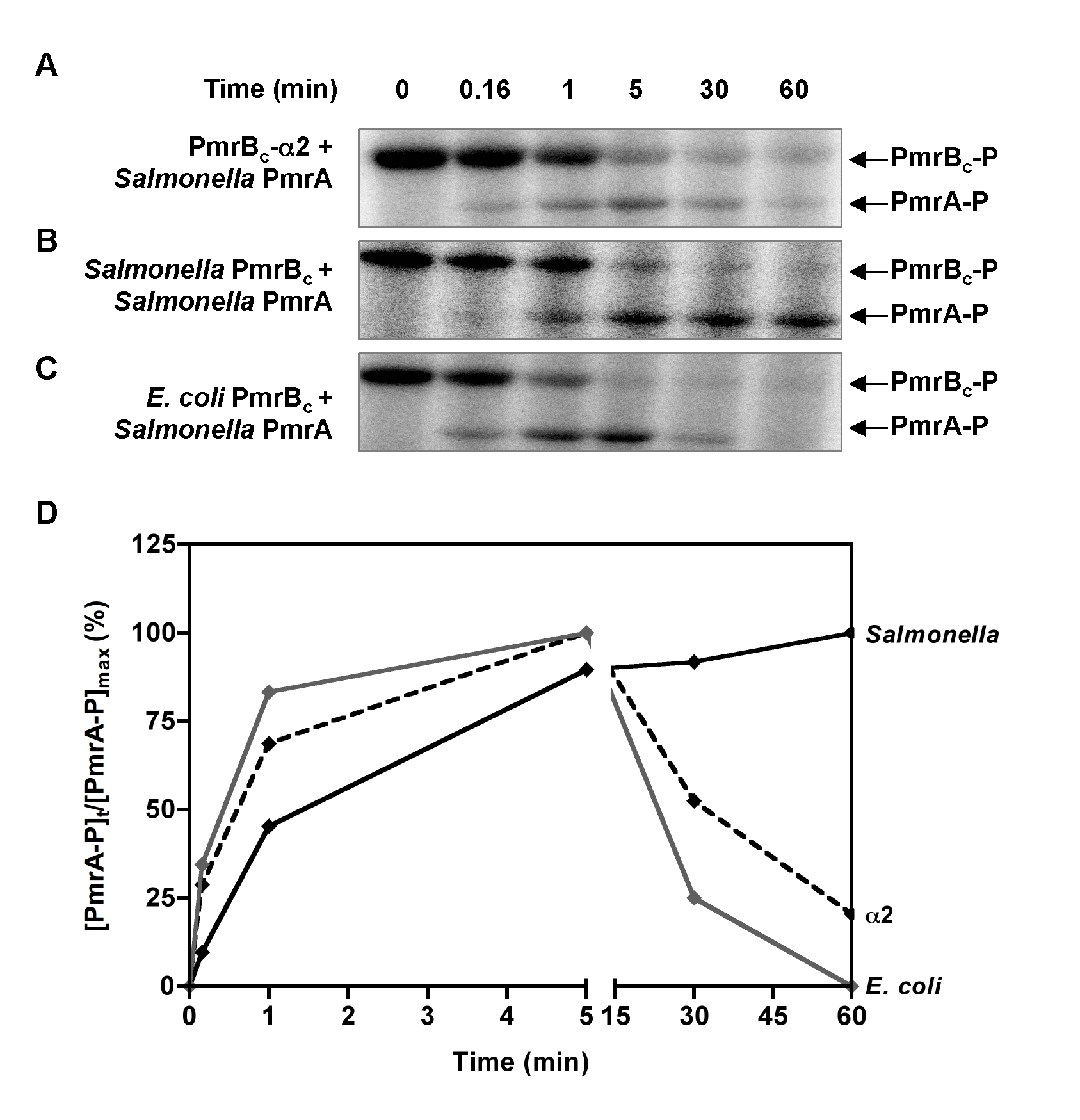

Supplement: Figure S6 — Phosphotransfer from the PmrBc-α2 chimera to the Salmonella PmrA protein is similar to that displayed by the E. coli PmrBc protein. (A–C) Levels of PmrBc-P and PmrA-P following incubation of PmrBc-P (5 µM) and PmrA (10 µM) as described in Text S1 with aliquots taken at the times indicated at the top of the figure. Three different reactions were set up with the PmrBc-α2 and Salmonella PmrA proteins (A), Salmonella PmrBc and Salmonella PmrA proteins (B), and E. coli PmrBc and Salmonella PmrA proteins (C). (D) Quantitation of the phosphotransfer assays shown in (A–C). The plot depicts the level of PmrA-P relative to the maximum achieved as a function of time. (TIF) [file pgen.1002184.s006.tif]

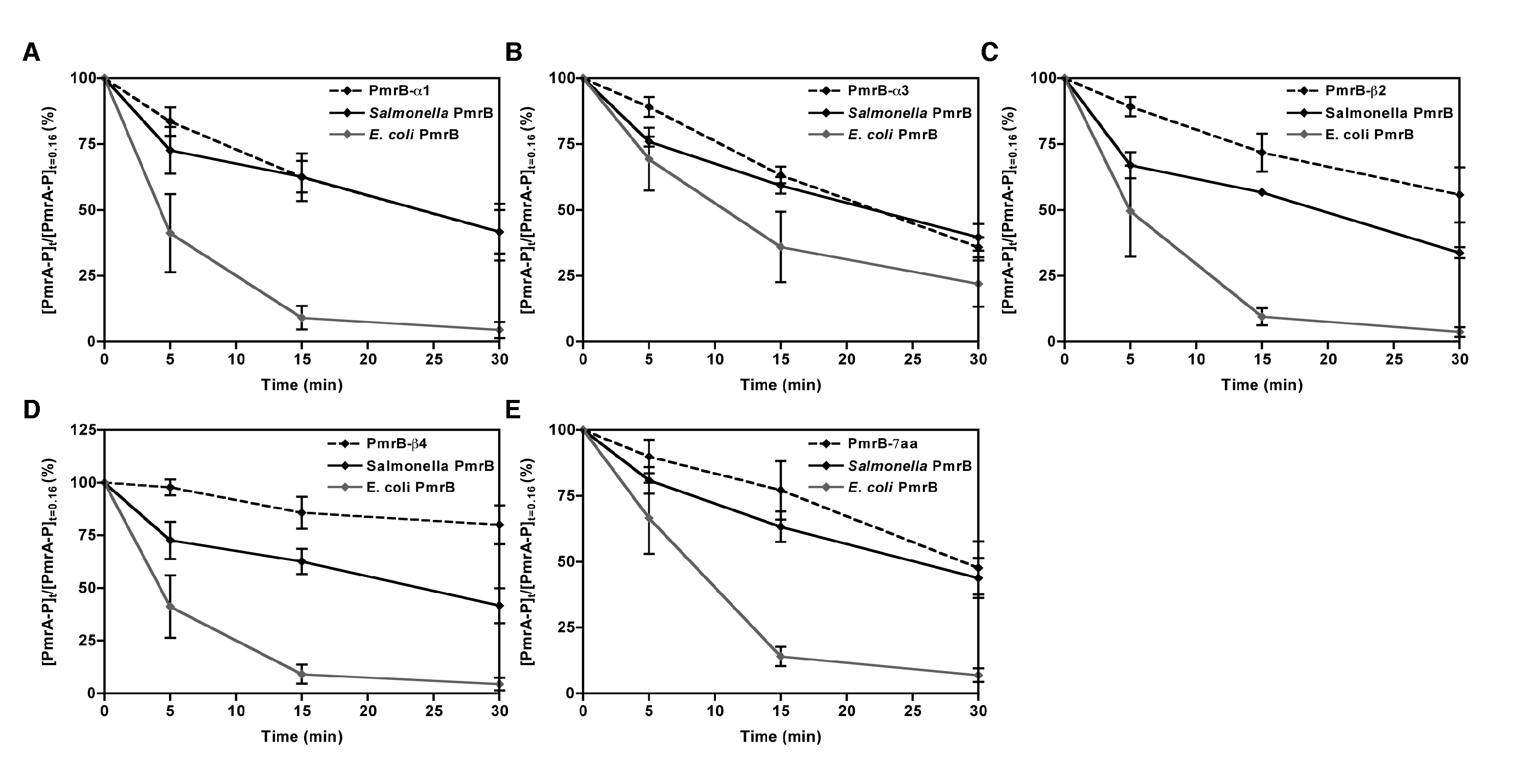

Supplement: Figure S7 — The PmrBc chimeras exhibit similar or lower levels of phosphatase activity than the Salmonella PmrBc protein. (A–E) The graphs depict the levels of PmrA-P at the indicated times relative to levels at the start of the reaction (t = 0.16 min) following incubation of Salmonella PmrA-P (2.5 µM) with the PmrBc-α1 protein (A) or PmrBc-α3 protein (B) or the PmrBc-β2 protein (C) or the PmrBc-β4 protein (D) or the PmrBc-7aa protein (E). The abilities of the PmrBc chimeric proteins (5 µM) to dephosphorylate PmrA-P were compared to those of the Salmonella or E. coli PmrBc proteins. Data correspond to the mean values of at least three independent experiments and error bars show standard deviation. (TIF) [file pgen.1002184.s007.tif]

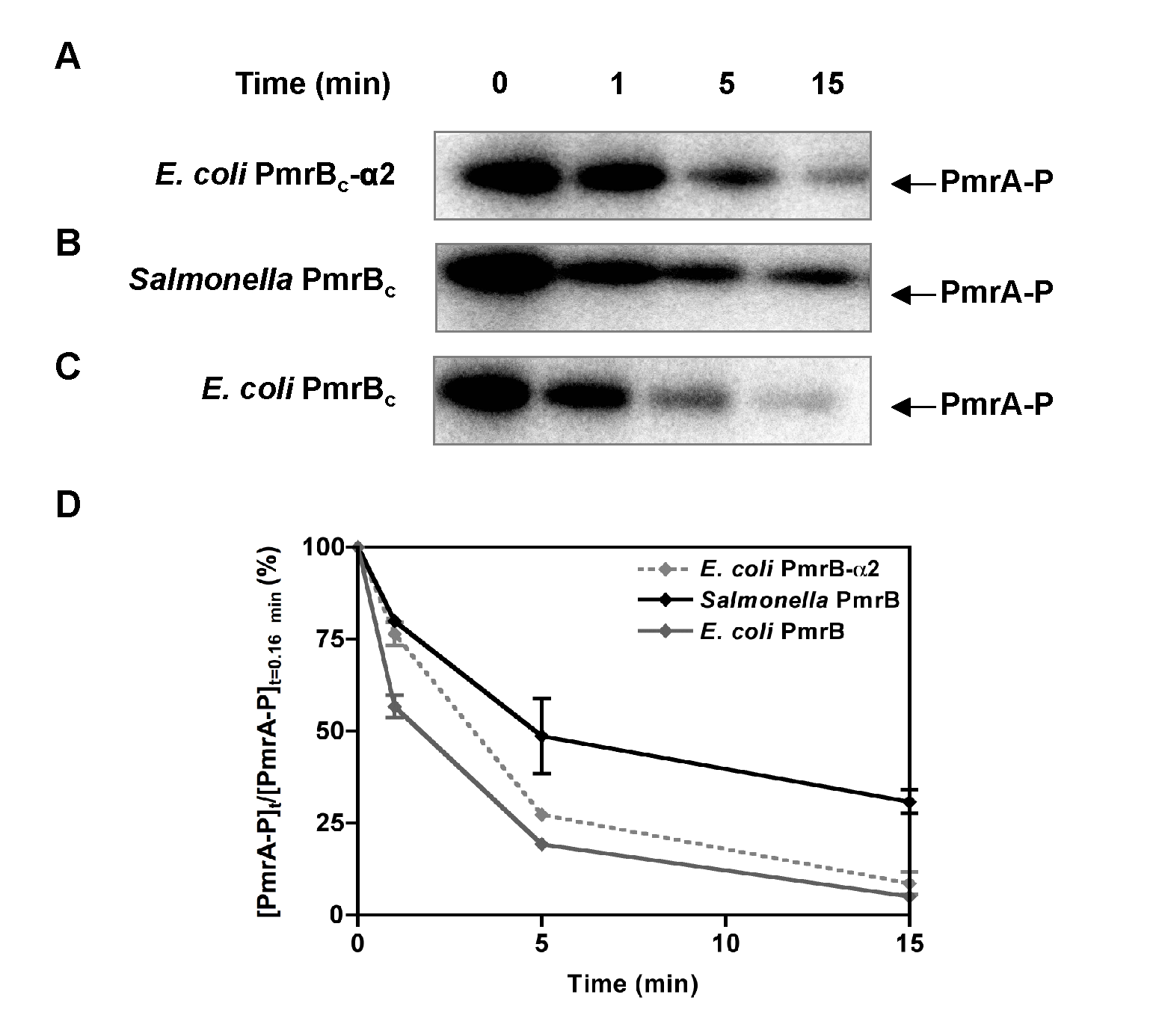

Supplement: Figure S8 — The E. coli PmrBc–α2 chimera exhibits a lower level of phosphatase activity than the E. coli PmrBc protein. (A–C) Levels of PmrA-P following incubation of E. coli PmrA-P (2.5 µM) with the E. coli PmrBc-α2 chimera (A), the Salmonella PmrBc (B) or the E. coli PmrBc (C) (5 µM) proteins for the indicated times. (D) Quantitation of the phosphatase assays shown in (A–C). The graph depicts the levels of PmrA-P at the indicated times relative to levels at the start of the reaction (t = 0.16 min). Data correspond to the mean values of three independent experiments and error bars show standard deviation. (TIF) [file pgen.1002184.s008.tif]

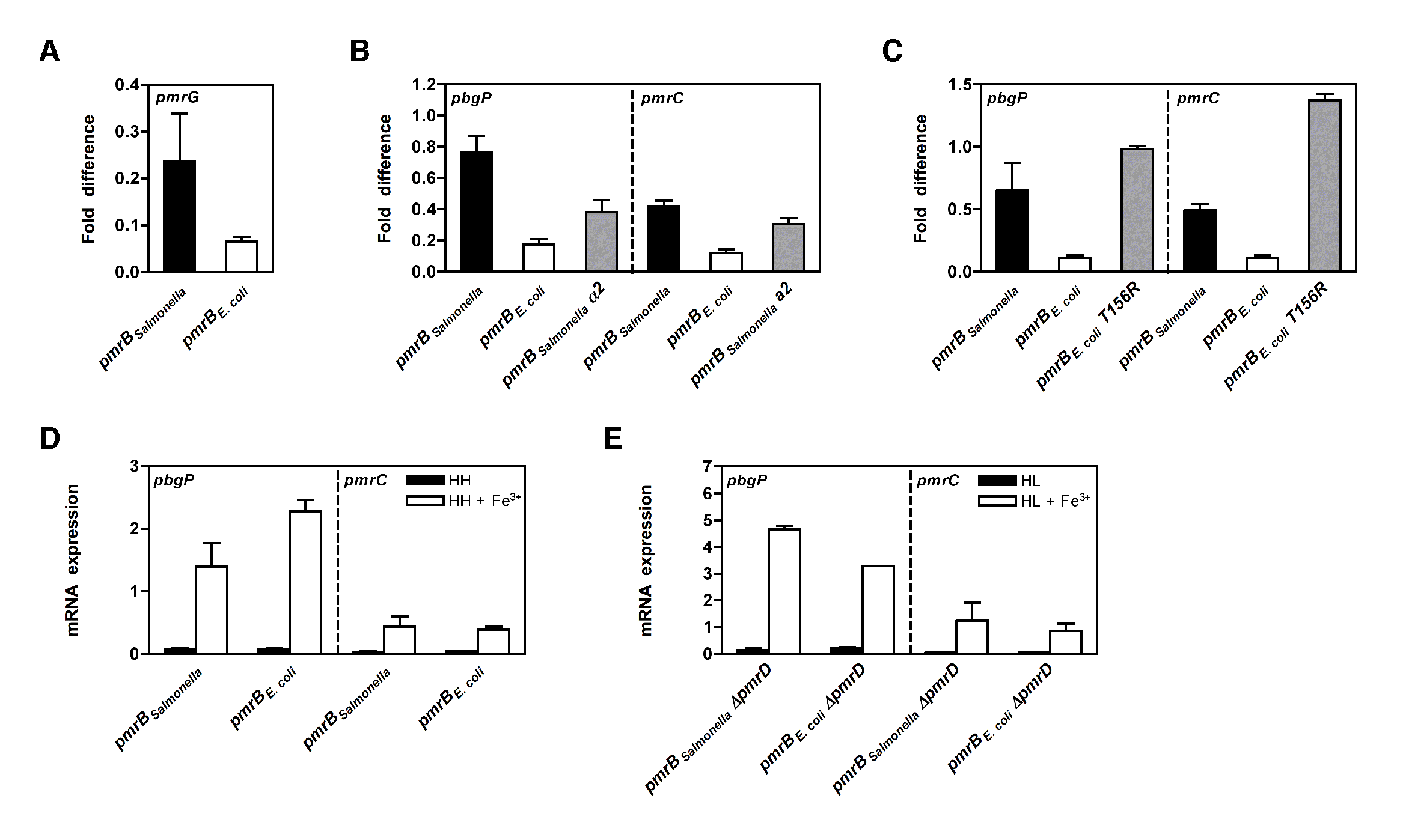

Supplement: Figure S9 — The Salmonella pmrB gene restores low Mg2+-promoted transcription of PmrA-activated genes in E. coli. (A–C) The ratio between the mRNA levels of the PmrA-activated genes pmrG, pmrC and pbgP produced during growth in low Mg2+ and those produced following growth in the presence of Fe3+ was determined in E. coli expressing the Salmonella pmrB gene (DC3) or its own pmrB gene (DC5) or the Salmonella pmrB-α2 gene (DC68) or the E. coli pmrB T156R gene (DC116) from the normal chromosomal location. RNA samples were prepared from bacteria grown in N-minimal media, pH 7.7 with 10 µM Mg2+ (HL) or 10 µM Mg2+ and 100 µM Fe3+ (HL+Fe3+) to OD600 of 0.5, and the levels of pmrG, pmrC or pbgP mRNA were determined by reverse-transcription-qPCR analysis. (D) Transcript levels of the PmrA-activated genes pbgP and pmrC were determined in E. coli strains expressing either the Salmonella pmrB gene (DC3) or the E. coli pmrB gene (DC5). Bacteria were grown in N-minimal media, pH 7.7 with 1 mM Mg2+ (HH) or 1 mM Mg2+ and 100 µM Fe3+ (HH+Fe3+) to an OD600 0.5 before RNA samples were prepared for reverse-transcription-qPCR analysis. (E) Transcript levels of the PmrA-activated genes pbgP and pmrC were determined in E. coli strains deleted for the pmrD gene and expressing either the Salmonella pmrB gene (DC9) or the E. coli pmrB gene (DC11). Bacteria were grown in N-minimal media, pH 7.7 with 10 µM Mg2+ (HL) or 10 µM Mg2+ and 100 µM Fe3+ (HL+Fe3+) to an OD600 0.5 before RNA samples were prepared for reverse-transcription-qPCR analysis. Data correspond to the mean of at least three independent experiments and error bars represent standard deviation. (TIF) [file pgen.1002184.s009.tif]
