# Supplementary material for: Ancestral Genes Can Control the Ability of Horizontally Acquired Loci to Confer New Traits
Source: PLoS Genet. 2011 Jul 21;7(7):e1002184. doi: 10.1371/journal.pgen.1002184 (PMC3140997; doi:10.1371/journal.pgen.1002184)
Supplement: Table S1 — McDonald-Kreitman test for pmrB evolution. (DOC) [file pgen.1002184.s010.doc]

**Table S1. McDonald-Kreitman test for *pmrB* evolution**

|  | **Fixed between species** | **Polymorphic within species** |
| --- | --- | --- |
| **Non-synonymous substitutions** | 58 | 24 |
| **Synonymous substitutions** | 159 | 115 |

Fisher’s exact test, P-value (two-tailed): 0.039928* (* 0.01<P<0.05)

n= 63 *E. coli*, n= 8 *Salmonella*
