# Supplementary material for: Ancestral Genes Can Control the Ability of Horizontally Acquired Loci to Confer New Traits
Source: PLoS Genet. 2011 Jul 21;7(7):e1002184. doi: 10.1371/journal.pgen.1002184 (PMC3140997; doi:10.1371/journal.pgen.1002184)
Supplement: Table S2 — Bacterial strains and plasmids used in this study. (DOC) [file pgen.1002184.s011.doc]

**Table S2. Bacterial strains and plasmids used in this study**

| **Strain or plasmid** | **Description** | **Reference or source** |
| --- | --- | --- |
| ***Salmonella* serovar Typhimurium** | | |
| 14028s | Wild-type | [1] |
| MS7953 | *phoP7953*::Tn*10* | [1] |
| EG9241 | *pbgP*:: MudJ | [2] |
| EG10065 | *pbgP*:: MudJ Δ*pmrB*::Cm^R^ | [5] |
| EG11491 | Δ*pmrD*::Cm^R^ | [3] |
| EG11775 | *pbgP*:: MudJ Δ*pmrD*::Cm^R^ | [3] |
| EG12060 | *pbgP*:: MudJ Δ*pmrB*::Cm^R^ Δ*pmrD*::Cm^R^ | [5] |
| EG13404 | *pmrD_Salmonella_^+^*-FLAG-Cm^R^ | [4] |
| EG13623 | *pmrD_E. coli_^+^*-FLAG-Cm^R^ | This work |
| EG13941 | *pbgP*:: MudJ *pmrD_E. coli_^+^*-FLAG-Cm^R^ | This work |
| EG13942 | *pbgP*:: MudJ *pmrD_Salmonella_^+^*-FLAG-Cm^R^ | This work |
| ***E. coli*** |  |  |
| MG1655 | Wild-type | [6] |
| DH5α | F–*sup*E44 Δ*lac*U169 (*φ* 80 *lacZ*ΔM15) *hsd*R17 *rec*A1 *end*A1 *gyr*A96 *thi*-1 *rel*A1 | [7] |
| ER2566 | *fhuA2* [*lon*] *ompT lacZ*::T7 *gene1 gal sulA11* Δ(*mcrC-mrr*)*114*::*IS10* R(*mcr-73*::miniTn*10*-Tet^S^)2 R(*zgb-210*::Tn*10*-Tet^S^) *endA1* [*dcm*] | New England Biolabs |
| EG13796 | ER2566 Δ*basRS*::Cm^R^ | [5] |
| EG15041 | *pmrD_E. coli_^+^*-FLAG-Cm^R^ | [8] |
| DC1 | Δ*pmrB*::TetA | This work |
| DC3 | *pmrB_Salmonella_*^+^-Cm^R^ | This work |
| DC5 | *pmrB_E coli._* ^+^-Cm^R^ | This work |
| DC9 | Δ*pmrD*::Cm^R^ *pmrB_Salmonella_*^+^-Cm^R^ | This work |
| DC11 | Δ*pmrD*::Cm^R^ *pmrB_E coli._* ^+^-Cm^R^ | This work |
| DC68 | *pmrB_Salmonella_-α2*^+^-Cm^R^ | This work |
| DC116 | *pmrB_E coli_. T156R*^+^-Cm^R^ | This work |
| DC121 | Δ*pmrD*::Cm^R^ *pmrB_E coli_. T156R*^+^-Cm^R^ | This work |
| **Plasmid** |  |  |
| pKD3 | rep_R6Kγ_ Ap^R^ FRT Cm^R^ FRT | [9] |
| pKD46 | rep_pSC101_ts Ap^R^ P*_araBAD_* γ β exo | [9] |
| pT7-7 | rep_PMB1_ Ap^R^ p_T7_ | [10] |
| pT7-7-PmrA*_Salmonella_*-His6 | rep_pMB1_ Ap^R^ p_T7_ *pmrA_Salmonella_*-His6 | [11] |
| pT7-7-PmrA*_E. coli_*-His6 | rep_pMB1_ Ap^R^ p_T7_ *pmrA_E. coli_* -His6 | This work |
| pT7-7-His6-PmrB_c_*_Salmonella_* | rep_pMB1_ Ap^R^ p_T7_ His6-*pmrB_cSalmonella_* | [11] |
| pT7-7-His6-PmrB_c_*_E. coli_* | rep_pMB1_ Ap^R^ p_T7_ His6- *pmrB_cE. coli_* | This work |
| pGEX-PmrB_c_*_Salmonella_*T156R | rep_pMB1_ Ap^R^ p*_tac_* GST-*pmrB_cSalmonella_T156R* | [5] |
| pT7-7-His6- PmrB_c_-α1 _a_ | rep_pMB1_ Ap^R^ p_T7_ His6-*pmrB_c_-α1* | This work |
| pT7-7-His6- PmrB_c_-α2 | rep_pMB1_ Ap^R^ p_T7_ His6-*pmrB_c_-α2* | This work |
| pT7-7-His6- PmrB_c_-α3 | rep_pMB1_ Ap^R^ p_T7_ His6-*pmrB_c_-α3* | This work |
| pT7-7-His6- PmrB_c_-β2 | rep_pMB1_ Ap^R^ p_T7_ His6-*pmrB_c_-β2* | This work |
| pT7-7-His6- PmrB_c_-β4 | rep_pMB1_ Ap^R^ p_T7_ His6-*pmrB_c_-β4* | This work |
| pT7-7-His6- PmrB_c_-7aa | rep_pMB1_ Ap^R^ p_T7_ His6-*pmrB_c_-7aa* | This work |
| pT7-7-  His6- PmrB_c_*_E. coli_*-α2 | rep_pMB1_ Ap^R^ p_T7_ His6-*pmrB_cE. coli_-α2* | This work |
| pT-7-7-His6-PmrB_c_T156R | rep_pMB1_ Ap^R^ p_T7_ His6-*pmrB_c_T156R* | This work |
| pUHE21-2*lacI^q^* | rep_pMB1_ Ap^R^ lacI^q^ | [12] |
| pUH-pmrD*_E. coli_* | rep_pMB1_ Ap^R^ lacI^q^ *pmrD­_E. coli_* | This work |
| pUH-pmrD*_E. coli_*-FLAG | rep_pMB1_ Ap^R^ lacI^q^ *pmrD­_E. coli_-FLAG* | This work |
| pUH-pmrB*_E. coli_* | rep_pMB1_ Ap^R^ lacI^q^ *pmrB­_E. coli_* | This work |
| pUH-pmrB_c_*_E. coli_* | rep_pMB1_ Ap^R^ lacI^q^ *pmrB_c_­_E. coli_* | This work |

**References**

1. Fields PI, Swanson RV, Haidaris CG, Heffron F (1986) Mutants of *Salmonella typhimurium* that cannot survive within the macrophage are avirulent. Proc Natl Acad Sci U S A 83: 5189-5193.

2. Soncini FC, Garcia Vescovi E, Solomon F, Groisman EA (1996) Molecular basis of the magnesium deprivation response in *Salmonella typhimurium*: identification of PhoP-regulated genes. J Bacteriol 178: 5092-5099.

3. Kox LF, Wosten MM, Groisman EA (2000) A small protein that mediates the activation of a two-component system by another two-component system. EMBO J 19: 1861-1872.

4. Kato A, Latifi T, Groisman EA (2003) Closing the loop: the PmrA/PmrB two-component system negatively controls expression of its posttranscriptional activator PmrD. Proc Natl Acad Sci U S A 100: 4706-4711.

5. Kato A, Groisman EA (2004) Connecting two-component regulatory systems by a protein that protects a response regulator from dephosphorylation by its cognate sensor. Genes Dev 18: 2302-2313.

6. Blattner FR, Plunkett G, 3rd, Bloch CA, Perna NT, Burland V, et al. (1997) The complete genome sequence of *Escherichia coli* K-12. Science 277: 1453-1462.

7. Hanahan D (1983) Studies on transformation of *Escherichia coli* with plasmids. J Mol Biol 166: 557-580.

8. Winfield MD, Groisman EA (2004) Phenotypic differences between *Salmonella* and *Escherichia coli* resulting from the disparate regulation of homologous genes. Proc Natl Acad Sci U S A 101: 17162-17167.

9. Datsenko KA, Wanner BL (2000) One-step inactivation of chromosomal genes in *Escherichia coli* K-12 using PCR products. Proc Natl Acad Sci U S A 97: 6640-6645.

10. Tabor S, Richardson CC (1985) A bacteriophage T7 RNA polymerase/promoter system for controlled exclusive expression of specific genes. Proc Natl Acad Sci U S A 82: 1074-1078.

11. Wosten MM, Groisman EA (1999) Molecular characterization of the PmrA regulon. J Biol Chem 274: 27185-27190.

12. Soncini FC, Vescovi EG, Groisman EA (1995) Transcriptional autoregulation of the Salmonella typhimurium phoPQ operon. J Bacteriol 177: 4364-4371
