# Supplementary material for: Ancestral Genes Can Control the Ability of Horizontally Acquired Loci to Confer New Traits
Source: PLoS Genet. 2011 Jul 21;7(7):e1002184. doi: 10.1371/journal.pgen.1002184 (PMC3140997; doi:10.1371/journal.pgen.1002184)
Supplement: Table S3 — DNA sequences of primers used in this study. (DOC) [file pgen.1002184.s012.doc]

**Table S3. DNA sequences of primers used in this study**

| **Primer number** | **Sequence 5’ 🡪 3’** |
| --- | --- |
| **Real-time PCR primers** | |
| 3023 | CCAGCAGCCGCGGTAAT |
| 3024 | TTTACGCCCAGTAATTCCGATT |
| 9525 | GCGTATTGAAGACGGGATCAA |
| 9526 | GATCGTACTGTTCCAGCAGATAGGT |
| 9643 | TCACAACCGGTCCGAAGAA |
| 9644 | TCCCGTCAACTGGCAAAAAG |
| 10035 | AGCCACGGTCCGACCTATTA |
| 10036 | ATCTCATTGGTGTCGCAGGTT |
| 10467 | CGTTTGTCTCGACCCACGTA |
| 10468 | CGGCAGACAATAACCACCATAA |
| 10469 | CCGCTCTTCGTTGAAGTCAAA |
| 10470 | TGAGTACCCAGTCCGGCAAT |

| **Cloning primers** | |
| --- | --- |
| 988 | AGATATACATATGCACCACCACCACCACCACCGGCGTATTACCCGTCCGCT |
| 1959 | ACGAAGCTTATGCCTTTTTCA |
| 1984 | GCATTCGCACGGTTCGCGGG |
| 2132 | TATTATGGCGGGGGTAATGCTGATTTTTCTGCCCGCCAGAGTGTAGGCTGGAGCTGCTTTC |
| 2127 | CATATGAATATCCTCCTTA |
| 2135 | TATTATGGCGGGGGTAATGCTGAT |
| 2136 | TGTAACGTGTTATGAACAATCAGC |
| 2284 | TGTAACGTGTTATGAACAATCAGCGTGAAACGGGGGCGCTATGGAATGGCTGGTCAAAAA |
| 2316 | TAAGGAGGATATTCATATGTCACTTGTCATCGTCGTCCTTGTAGTCCTGAGTTTTCCCTGCCA |
| 2449 | GAGGATCCATATGAAAATTCTGATTG |
| 2461 | TCCAAGCTTAGTGGTGGTGGTGGTGGTGGTTTTCCTCATTCGCGA |
| 2447 | GGTGGTTGCTCTTCCAACATGGAATGGCTGGTCA |
| 2448 | ACCTGCAGTCACTTGTCATCGTCGTCCTTGTAGTCCTGAGTTTTCCCTGCCACT |
| 8895 | AGATATACATATGCACCACCACCACCACCACCGCCGCATCACCCGCCCGCT |
| 8896 | ACGAAG CTTATATCTGGTTTGCCACGT |
| 9094 | GTCAGGATCCATGGAATGGCTGGTCAAAAA |
| 9110 | GTCAAAGCTT TTACTGAGTTTTCCCTGCCA |
| 9214 | GTCAGGATCCTCACTTGTCATCGTCGTCCTTGTAGTC CTGAGTTTTCCCTGCCA |
| 9390 | GCCAGTGGCCGTCGCAGTTCGTGCGCGA |
| 9391 | TCGCGCACGAACTGCGACGGCCACTGGC |
| 9827 | CTATATGCTGGTCGCGAATGAGGAAAACTAATTGAATCTGCTCTAATGCGCTGTTAATCACT |
| 9831 | GTTTAACTACCGTGTTCAGCGTGCTGGTGGTCAGCAGCTTTCTCTAAGCACTTGTCTCCTGTT |
| 9840 | GTGCATATCCACAATCTGCG |
| 9841 | GAAACGTACCCGCCTGGT |
| 9842 | CGATCAGGAAATTGCGGAGC |
| 9843 | CTATATGCTGGTCGCGAATGAGGAAAACTAATTGAATCTGATGCGTTTTCAGCGAAGAGCG |
| 10270 | AGGAGAGTGCAATGAAAAACCGTGTTTATGAAAGTTTAACTACCGTGTTCAGCGTGTGTAGGCTGGAGCTGCTTC |
| 10306 | GTTGAAAAAGGCATAAAGAAAGCTGCTGACCACCAGCCATATGAATATCCTCCTTAG |
| 10308 | GTGCAATGAAAAACCGTGTTTATGAAAGTTTAACTACCGTGTTCAGCGTGTGTAGGCTGGAGCTGCTTC |
| 10305 | GAAGAAAGATCAGTACGTGGCAAACCAGATATAAAGAAAGCTGCTGACCACCAGCCATATGAATATCCTCCTTAG |
| 10311 | TATTCATATGGCTGGTGGTCAGCAGCTTTCT TTATGCCTTTTTCAACAGCACCCAG |
| 10677 | GTCGAGCATGGTACTCAGCTCATCGTAGGAGGGGAGGATC |
| 10678 | GCTGAGTACCATGCTCGACCAGCGCCAGCAAACTCTGTTGCTG |
| 10679 | GACGTCAGCGGTAAACAACCTTTCGTTATCCAGCGTGCTGGTCAGGCGACTGACCAGATCGTTTAACGCCGAGACGACGGACTCAATCTC |
| 10687 | GGTTGTTTACCGCTGACGTCGCGCACGAACTGCGAACGCCACTGGCGGGGGTGCGTTTGCATCTGGAACTGCTGGCGAAAACCCACAATGTTGATGTC |
| 10680 | GACGCTCTCCATCATCTGATCAAGCCGTGCCACTAACGGTGCTACATCAATGTGGTGGGTTTTTGACAATAATTCCAG |
| 10681 | ATCAGATGATGGAGAGCGTCTCCCAGCTGCTGCAACTGGCGCGTGCCGGACAGTCATTCTCTTCCGGGAATTATCAG |
| 10682 | TCCGTCATCTTCTTGCAGCTTAATCATAATGTTGCTGCCTTGAGGGCTATAGCGATGCGCGTTTTC |
| 10683 | CAAGGCAGCAACATTATGATTAAGCTGCAAGAAGATGACGGAGCTATTATGGCGGTCGAAGACG |
| 10684 | GTGCCGGAAGTCTCTTGCCGGTTTTGCAGGAAAAACTGCCCGTGATGTAGTTGGGTGATGCGGCTG |
| 10685 | CCCAAGCTTTTATGCCTTTTTCAGCCGTACCCAGGCCCGCGTGCCGGAAGTCTCTTGCCG |
| 10686 | CCCAAGCTTTTATATCTGGTTTGCCACGTACTGATCCTTTTTCAACAGCACCCAGGCACGG |
| 11244 | CCCACAATATTGATGTCGCG |
| 11245 | CGCGACATCAATATTGTGGG |
| 11250 | CAACTGGCGCGCGCCGGCCAGTCATTC |
| 11251 | GAATGACTGGCCGGCGCGCGCCAGTTG |
| 11631 | GACGCTATCCATCATCTGGTCAAGACGGGCGATAAGCGGCGCGACATCAACATTATGCGTTTTCGCCAGCAGTTC |
| 11632 | ACCAGATGATGGATAGCGTCTCCCAGCTTCTGCAACTGGCGCGCGTGGGCCAGTCATTTTCTTCCGGTAATTATC |
| 11634 | TTATATCTGGTTTGCCACGTAC |
| 11635 | CGCGGCTTTGGCTATATGCTGGTCGCGAATGAGGAAAACTAATTGAATCTG ATGCATTTTCTGCGCCGACC |
| 11863 | GTACGGATCCATGCATTTTCTGCGCCGAC |
| 11864 | GTACAAGCTTTTATATCTGGTTTGCCACG |
| 11865 | GTACGGATCCATGCGCCGCATCACCCGCCCGCT |
