# Supplementary material for: Ancestral Genes Can Control the Ability of Horizontally Acquired Loci to Confer New Traits
Source: PLoS Genet. 2011 Jul 21;7(7):e1002184. doi: 10.1371/journal.pgen.1002184 (PMC3140997; doi:10.1371/journal.pgen.1002184)
Supplement: Table S4 — GenBank accession numbers for the pmrB genes from Salmonella and E. coli natural isolates. (DOC) [file pgen.1002184.s013.doc]

**Table S4. GenBank accession numbers for the *pmrB* genes from *Salmonella* and *E. coli* natural isolates**

| **Sequence_ID** | **Isolate** | **Accession number** |
| --- | --- | --- |
| ***E. coli*** | | |
| ECOR16_pmrB | ECOR16 | JN032018 |
| ECOR14_pmrB | ECOR14 | JN032019 |
| ECOR4_pmrB | ECOR4 | JN032020 |
| ECOR13_pmrB | ECOR13 | JN032021 |
| ECOR7_pmrB | ECOR7 | JN032022 |
| ECOR70_pmrB | ECOR70 | JN032023 |
| ECOR8_pmrB | ECOR8 | JN032024 |
| ECOR48_pmrB | ECOR48 | JN032025 |
| ECOR69_pmrB | ECOR69 | JN032026 |
| ECOR28_pmrB | ECOR28 | JN032027 |
| ECOR19_pmrB | ECOR19 | JN032028 |
| ECOR20_pmrB | ECOR20 | JN032029 |
| ECOR56_pmrB | ECOR56 | JN032030 |
| ECOR66_pmrB | ECOR66 | JN032031 |
| ECOR65_pmrB | ECOR65 | JN032032 |
| ECOR41_pmrB | ECOR41 | JN032033 |
| ECOR53_pmrB | ECOR53 | JN032034 |
| ECOR59_pmrB | ECOR59 | JN032035 |
| ECOR55_pmrB | ECOR55 | JN032036 |
| ECOR57_pmrB | ECOR57 | JN032037 |
| ECOR67_pmrB | ECOR67 | JN032038 |
| ECOR6_pmrB | ECOR6 | JN032039 |
| ECOR30_pmrB | ECOR30 | JN032040 |
| ECOR33_pmrB | ECOR33 | JN032041 |
| ECOR34_pmrB | ECOR34 | JN032042 |
| ECOR68_pmrB | ECOR68 | JN032043 |
| ECOR71_pmrB | ECOR71 | JN032044 |
| ECOR10_pmrB | ECOR10 | JN032045 |
| ECOR31_pmrB | ECOR31 | JN032046 |
| ECOR43_pmrB | ECOR43 | JN032047 |
| ECOR37_pmrB | ECOR37 | JN032048 |
| ECOR42_pmrB | ECOR42 | JN032049 |
| ECOR26_pmrB | ECOR26 | JN032050 |
| ECOR27_pmrB | ECOR27 | JN032051 |
| ECOR45_pmrB | ECOR45 | JN032052 |
| ECOR61_pmrB | ECOR61 | JN032053 |
| ECOR62_pmrB | ECOR62 | JN032054 |
| ECOR58_pmrB | ECOR58 | JN032055 |
| ECOR24_pmrB | ECOR24 | JN032056 |
| ECOR72_pmrB | ECOR72 | JN032057 |
| ECOR29_pmrB | ECOR29 | JN032058 |
| ECOR9_pmrB | ECOR9 | JN032059 |
| ECOR25_pmrB | ECOR25 | JN032060 |
| ECOR21_pmrB | ECOR21 | JN032061 |
| ECOR23_pmrB | ECOR23 | JN032062 |
| MG1655_pmrB | MG1655 | JN032063 |
| ECOR1_pmrB | ECOR1 | JN032064 |
| ECOR12_pmrB | ECOR12 | JN032065 |
| ECOR15_pmrB | ECOR15 | JN032066 |
| ECOR5_pmrB | ECOR5 | JN032067 |
| ECOR18_pmrB | ECOR18 | JN032068 |
| ECOR17_pmrB | ECOR17 | JN032069 |
| ECOR46_pmrB | ECOR46 | JN032070 |
| ECOR35_pmrB | ECOR35 | JN032071 |
| ECOR36_pmrB | ECOR36 | JN032072 |
| ECOR38_pmrB | ECOR38 | JN032073 |
| ECOR39_pmrB | ECOR39 | JN032074 |
| ECOR47_pmrB | ECOR47 | JN032075 |
| ECOR44_pmrB | ECOR44 | JN032076 |
| ECOR54_pmrB | ECOR54 | JN032077 |
| ECOR52_pmrB | ECOR52 | JN032078 |
| ECOR32_pmrB | ECOR32 | JN032079 |
| ECOR51_pmrB | ECOR51 | JN032080 |
| ***S. enterica*** | | |
| SARA62_pmrB | SARA62 | JN040470 |
| SARA64_pmrB | SARA64 | JN040471 |
| s3333_pmrB | s3333 | JN040472 |
| s2985_pmrB | s2985 | JN040473 |
| s2995_pmrB | s2995 | JN040474 |
| s3057_pmrB | s3057 | JN040475 |
| s3027_pmrB | s3027 | JN040476 |
