# Supplementary material for: Ancestral Genes Can Control the Ability of Horizontally Acquired Loci to Confer New Traits
Source: PLoS Genet. 2011 Jul 21;7(7):e1002184. doi: 10.1371/journal.pgen.1002184 (PMC3140997; doi:10.1371/journal.pgen.1002184)
Supplement: Text S1 — Supplementary materials and methods. (DOC) [file pgen.1002184.s014.doc]

**Text S1. Supplementary materials and methods**

**Construction of *Salmonella* Chromosomal Mutants.** To construct an isogenic *Salmonella* derivative of strain 14028s harboring the *E. coli* *pmrD* coding region (EG13623), we used a modification of the one-step inactivation protocol [1]. DNA fragments encompassing the C-terminally FLAG-tagged *E. coli* *pmrD* coding regions and a Cm^R^ cassette were generated by performing two sequential PCR reactions. A C-terminally FLAG-tagged *E. coli* *pmrD* coding region was amplified from the *E. coli* chromosome using primers 2284 and 2316. The Cm^R^ cassette was amplified from the plasmid pKD3 using primers 2132 and 2127. A second PCR was performed using the first two amplicons as the template to combine the sequences encoding the C-terminally FLAG-tagged *E. coli pmrD* coding region and the Cm^R^ cassette with primers 2135 and 2136. The PCR products were purified using a QIAquick PCR purification kit (Qiagen) and used to electroporate *Salmonella* 14028s containing plasmid pKD46 [1] selecting for chloramphenicol-resistant transformants at 37˚C. Gene replacements were confirmed by sequence analysis, and expression of the *E. coli* PmrD-FLAG protein was confirmed by Western blot analysis using anti-FLAG antibodies (Sigma) as described [2].

**Construction of *E. coli* Chromosomal Mutants.** To construct an *E. coli* derivative of strain MG1655 harboring the *Salmonella pmrB* ORF (DC3), we used a modification of the one-step inactivation protocol [1]. A Tet^R^ cassette was amplified using primers 9827 and 9831 and genomic DNA from strain MS7953s, which harbors a Tn*10* transposon in the *phoP* gene [3]. The PCR product was gel purified and used to electroporate *E. coli* MG1655 containing plasmid pKD46 [1] selecting for tetracycline-resistant transformants at 30˚C. The resultant Δ*pmrB*::*tetA* strain (DC1) containing plasmid pKD46 was kept at 30°C. DNA fragments that encompassed the *Salmonella pmrB* ORF and a Cm^R^ cassette downstream of the *pmrB* gene were generated by performing two sequential PCR reactions. The *Salmonella pmrB* ORF was amplified with primers 9843 and 10311 using 14028s genomic DNA as a template; the Cm^R^ cassette was amplified from plasmid pKD3 using primers 10306 and 10308. A second PCR was performed using the first two amplicons as templates and primers 9843 and 10308. The PCR product was gel purified, electroporated into strain DC1 containing plasmid pKD46 to obtain chloramphenicol-resistant recombinants, which were then screened for tetracycline-sensitivity. Gene replacements were confirmed by sequence analysis, and PmrB protein expression was confirmed by Western blot analysis using anti-PmrB antibodies as described in [4].

To construct an *E. coli* derivative of strain MG1655 harboring the *Salmonella pmrB-α2* ORF (DC68), in which residues 173–201 of the *Salmonella* PmrB were replaced by those corresponding to the *E. coli* PmrB protein, we used a modification of the one-step inactivation protocol [1]. DNA fragments that encompassed the *Salmonella pmrB-α2* ORF and a Cm^R^ cassette downstream of the *pmrB* gene were generated by performing two sequential PCR reactions. The *Salmonella pmrB-α2* ORF was amplified with primers 9843 and 10680 and with 10311 and 10687 using 14028s genomic DNA as a template; the Cm^R^ cassette was amplified from plasmid pKD3 using primers 10306 and 10308. A fourth PCR was performed using the first three amplicons as templates and primers 9843 and 10308. The PCR product was gel purified, electroporated into strain DC1 containing plasmid pKD46 to obtain chloramphenicol-resistant recombinants, which were then screened for tetracycline-sensitivity. Gene replacements were confirmed by sequence analysis.

To construct an *E. coli* derivative of strain MG1655 harboring the *E. coli pmrB T156R* gene (DC116), we used a modification of the one-step inactivation protocol [1]. DNA fragments that encompassed the *E. coli pmrB T156R* gene and a Cm^R^ cassette downstream of the *pmrB* gene were generated by performing two sequential PCR reactions. The *E. coli pmrB T156R* gene was amplified with primers 11635 and 9390 and with 11632 and 9391 using MG1655 genomic DNA as a template; the Cm^R^ cassette was amplified from plasmid pKD3 using primers 10305 and 10270. A fourth PCR was performed using the first three amplicons as templates and primers 10270 and 11635. The PCR product was gel purified, electroporated into strain DC1 containing plasmid pKD46 to obtain chloramphenicol-resistant recombinants, which were then screened for tetracycline-sensitivity. Gene replacements were confirmed by sequence analysis.

To construct an *E. coli* derivative of strain MG1655 harboring a Cm^R^ insertion downstream of the *pmrB* gene (DC5), we used a modification of the one-step inactivation protocol [1]. A Cm^R^ cassette was amplified from plasmid pKD3 using primers 10305 and 10270. The PCR product was gel purified and electroporated into *E. coli* MG1655 containing plasmid pKD46 [1] selecting for chloramphenicol-resistant transformants at 37˚C. Gene replacements were confirmed by sequence analysis, and PmrB protein expression was confirmed by Western blot analysis as described in [4].

**Plasmid Constructions.** Plasmid pUH-pmrD*_E. coli_* encoding the *E. coli pmrD* coding region was constructed by cloning a PCR fragment generated with primers 9094 and 9110 and MG1655 DNA as a template between the *BamHI* and *HindIII* sites of plasmid pUHE21-2*lacI^q^*.

Plasmid pUH-pmrD*_E. coli_*-FLAG encoding the *E. coli pmrD* with a FLAG tag at the C-terminus was constructed by cloning a PCR fragment generated with primers 9094 and 9214 and MG1655 DNA as a template between the *BamHI* and *HindIII* sites of plasmid pUHE21-2*lacI^q^*.

Plasmid pUH-PmrB*_E. coli_* encoding the *E. coli pmrB* coding region was constructed by cloning a PCR fragment generated with primers 11863 and 11864 and MG1655 DNA as a template between the *BamHI* and *HindIII* sites of plasmid pUHE21-2*lacI^q^*.

Plasmid pUH-PmrB_c_*_E. coli_* encoding the *E. coli pmrB* cytoplasmic domain was constructed by cloning a PCR fragment generated with primers 11865 and 11864 and MG1655 DNA as a template between the *BamHI* and *HindIII* sites of plasmid pUHE21-2*lacI^q^*.

Plasmid pT-7-7-His6-PmrB_c_*_E. coli_* encoding the *E. coli pmrB* cytoplasmic domain with a His6 tag at the N-terminus was constructed by cloning a PCR fragment generated with primers 8895 and 8896 and MG1655 DNA as a template between the *Nde*I and *Hind*III sites of plasmid pT7-7.

Plasmid pT-7-7-His6-PmrB_c_*_E. coli_* T156R encoding the cytoplasmic domain of a mutant *E. coli* PmrB protein with the T156R substitution and a His6 tag at the N-terminus was constructed by using QuikChange site-directed mutagenesis kit (Stratagene) with pT-7-7-His6-PmrB_c_*_E. coli_* plasmid DNA as template and primers 9390 and 9391.

Plasmid pT-7-7-PmrA*_E. coli_*-His6 encoding the *E. coli pmrA* with a His6 tag at the C-terminus was constructed by cloning a PCR fragment generated with primers 2449 and 2461 and MG1655 DNA as a template between the *Nde*I and *Hind*III sites of plasmid pT7-7.

Plasmid pT-7-7-His6-PmrB_c_–α2 encodes the N-terminally His6-tagged *pmrB-α2* cytoplasmic domain, in which residues 173–201 of the *Salmonella* PmrB were replaced by those corresponding to the *E. coli* PmrB protein. DNA fragments encompassing the N-terminally His6-tagged *pmrB-α2* cytoplasmic domain were generated by performing two sequential PCR reactions. The N-terminally His6-tagged *Salmonella pmrB* coding region (corresponding to amino acids 89–172) was amplified from plasmid pT7-7-His6-PmrB_c_*_Salmonella_* using primer pairs 988 and 10680 and also harbored 54 bp of the *E. coli* *pmrB* coding region (corresponding to amino acids 173–190) on the 3’ end. The sequence corresponding to amino acids 202–356 of the *Salmonella pmrB* coding region was amplified from plasmid pT7-7-His6-PmrB_c_*_Salmonella_* using primer pairs 10687 and 1959 and also harbored 34 bp of the *E. coli* *pmrB* coding region (corresponding to amino acids 184–201) on the 5’ end. A second PCR was performed using the first two amplicons as the template to combine the sequences encoding the N-terminally His6-tagged *pmrB-α2* cytoplasmic domain using primers 988 and 1959. The PCR product was gel purified, digested with *NdeI* and *HindIII*, and cloned into plasmid pT7-7.

Plasmid pT-7-7-His6-PmrB_c_–α1 encodes the N-terminally His6-tagged *pmrB-α1* cytoplasmic domain, in which residues 127 - 169 of the *Salmonella* PmrB were replaced by those corresponding to the *E. coli* PmrB protein. DNA fragments encompassing the N-terminally His6-tagged *pmrB-α1* cytoplasmic domain were generated by performing two sequential PCR reactions. The N-terminally His6-tagged *Salmonella pmrB* coding region (corresponding to amino acids 89–126) was amplified from plasmid pT7-7-His6-PmrB_c_*_Salmonella_* using primer pairs 988 and 10679 and also harbored 68 bp of the *E. coli* *pmrB* coding region (corresponding to amino acids 127–150) on the 3’ end. The sequence corresponding to amino acids 170–356 of the *Salmonella pmrB* coding region was amplified from plasmid pT7-7-His6-PmrB_c_*_Salmonella_* using primer pairs 10687 and 1959 and also harbored 76 bp of the *E. coli* *pmrB* coding region (corresponding to amino acids 144–169) on the 5’ end. A second PCR was performed using the first two amplicons as the template to combine the sequences encoding the N-terminally His6-tagged *pmrB-α1* cytoplasmic domain using primers 988 and 1959. The PCR product was purified, digested with *NdeI* and *HindIII*, and cloned into plasmid pT7-7.

Plasmid pT-7-7-His6-PmrB_c_–α3 encodes the N-terminally His6-tagged *pmrB-α3* cytoplasmic domain, in which residues 225–230 of the *Salmonella* PmrB were replaced by those corresponding to the *E. coli* PmrB protein. DNA fragments encompassing the N-terminally His6-tagged *pmrB-α3* cytoplasmic domain were generated by performing two sequential PCR reactions. The N-terminally His6-tagged *Salmonella pmrB* coding region (corresponding to amino acids 89–224) was amplified from plasmid pT7-7-His6-PmrB_c_*_Salmonella_* using primer pairs 988 and 10677 and also harbored 14 bp of the *E. coli* *pmrB* coding region (corresponding to amino acids 225–229) on the 3’ end. The sequence corresponding to amino acids 231–356 of the *Salmonella pmrB* coding region was amplified from plasmid pT7-7-His6-PmrB_c_*_Salmonella_* using primer pairs 10678 and 1959 also harbored 17 bp of the *E. coli* *pmrB* coding region (corresponding to amino acids 225–230) on the 5’ end. A second PCR was performed using the first two amplicons as the template to combine the sequences encoding the N-terminally His6-tagged *pmrB-α3* cytoplasmic domain using primers 988 and 1959. The PCR product was purified, digested with *NdeI* and *HindIII*, and cloned into plasmid pT7-7.

Plasmid pT-7-7-His6-PmrB_c_–β2 encodes the N-terminally His6-tagged *pmrB-β2* cytoplasmic domain, in which residues 271 - 284 of the *Salmonella* PmrB were replaced by those corresponding to the *E. coli* PmrB protein. DNA fragments encompassing the N-terminally His6-tagged *pmrB-β2* cytoplasmic domain were generated by performing two sequential PCR reactions. The N-terminally His6-tagged *Salmonella pmrB* coding region (corresponding to amino acids 89–270) was amplified from plasmid pT7-7-His6-PmrB_c_*_Salmonella_* using primer pairs 988 and 10682 and also harbored 42 bp of the *E. coli* *pmrB* coding region (corresponding to amino acids 271–284) on the 3’ end. The sequence corresponding to amino acids 285–356 of the *Salmonella pmrB* coding region was amplified from plasmid pT7-7-His6-PmrB_c_*_Salmonella_* using primer pairs 10683 and 1959 and also harbored 42 bp of the *E. coli* *pmrB* coding region (corresponding to amino acids 271–284) on the 5’ end. A second PCR was performed using the first two amplicons as the template to combine the sequences encoding the N-terminally His6-tagged *pmrB-β2* cytoplasmic domain using primers 988 and 1959. The PCR product was purified, digested with *NdeI* and *HindIII*, and cloned into plasmid pT7-7.

Plasmid pT-7-7-His6-PmrB_c_–β4 encodes the N-terminally His6-tagged *pmrB-β4* cytoplasmic domain, in which residues 333–353 of the *Salmonella* PmrB were replaced by those corresponding to the *E. coli* PmrB protein. DNA fragments encompassing the N-terminally His6-tagged *pmrB-β4* cytoplasmic domain were generated by performing two sequential PCR reactions. The N-terminally His6-tagged *Salmonella pmrB* coding region (corresponding to amino acids 89–332) was amplified from plasmid pT7-7-His6-PmrB_c_*_Salmonella_* using primer pairs 988 and 10684 and also harbored 43 bp of the *E. coli* *pmrB* coding region (corresponding to amino acids 325–346) on the 3’ end. This amplicon was used as a template for a second PCR with primer pairs 988 and 10685 to generate sequences encoding the N-terminally His6-tagged *pmrB-β4* cytoplasmic domain. The PCR product was purified, digested with *NdeI* and *HindIII*, and cloned into plasmid pT7-7.

Plasmid pT-7-7-His6-PmrB_c_–7aa encodes the N-terminally His6-tagged *pmrB-7aa* cytoplasmic domain, in the *Salmonella* PmrB has an additional 7 amino acids on its C-terminus corresponding amino acids 356–363 of the *E. coli* PmrB protein. DNA fragments encompassing the N-terminally His6-tagged *pmrB-7aa* cytoplasmic domain were amplified from plasmid pT7-7-His6-PmrB_c_*_Salmonella_* using primer pairs 988 and 10686. The PCR product was purified, digested with *NdeI* and *HindIII*, and cloned into plasmid pT7-7.

Plasmid pT-7-7-His6-PmrB_c_*_E. coli_*–*α*2 encodes the N-terminally His6-tagged *pmrB-α2* cytoplasmic domain, in which residues 173–201 of the *E. coli* PmrB were replaced by those corresponding to the *Salmonella* PmrB protein. DNA fragments encompassing the N-terminally His6-tagged *pmrB-α2* cytoplasmic domain were generated by performing two sequential PCR reactions. The N-terminally His6-tagged *E.coli pmrB* coding region (corresponding to amino acids 89–172) was amplified from plasmid pT7-7-His6-PmrB_c_*_E.coli_* using primer pairs 8895 and 11631 and also harbored 53 bp of the *E. coli* *pmrB* coding region (corresponding to amino acids 173–190) on the 3’ end. The sequence corresponding to amino acids 202–356 of the *E. coli pmrB* coding region was amplified from plasmid pT7-7-His6-PmrB_c_*_E. coli_* using primer pairs 11631 and 8896 and also harbored 52 bp of the *Salmonella*  *pmrB* coding region (corresponding to amino acids 184–201) on the 5’ end. A second PCR was performed using the first two amplicons as the template to combine the sequences encoding the N-terminally His6-tagged *pmrB-α2* cytoplasmic domain using primers 8895 and 8896. The PCR product was gel purified, digested with *NdeI* and *HindIII*, and cloned into plasmid pT7-7.

PCR reactions were carried out with AccuPrime^TM^ Taq DNA Polymerase High Fidelity (Invitrogen), and the nucleotide sequences of the constructs were verified by DNA sequencing.

**Overproduction and Purification of Proteins.** The PmrA and PmrB_c_ proteins from *Salmonella* and from *E. coli* were overproduced in *E. coli* strain EG13796 harboring the following plasmids: pT7-7-PmrA*_Salmonella_*-His6, pT-7-7-His6-PmrB_c_*_Salmonella_*, pT7-7-PmrA*_E. coli_*-His6 or pT-7-7-His6-PmrB_c_*_E. coli_*. The PmrB_c_-α2 chimeric protein was overproduced in *E. coli* strain EG13796 harboring plasmid pT-7-7-His6-PmrB_c_–α2. All chimeric PmrB_c_ proteins were soluble when overproduced in *E. coli* and could be purified to similar levels as the original *Salmonella* and *E. coli* PmrB_c_ proteins. Cells were grown in 1 L of LB broth at 37°C to an OD_600_ of 0.5. Isopropyl-1-thio-β-D-galactopyranoside (IPTG) was added to a final concentration of 1 mM and incubation was continued for 16 h at 25°C. Cells were harvested and the pellet was stored overnight at -80°C. The pellet was resuspended in 10 ml of buffer containing 50 mM NaH_2_PO_4_ (pH 8.0), 300 mM NaCl and 10 mM imidazole and disrupted by sonication. Cell debris was pelleted by centrifugation at 10,000 rpm for 30 min. 3 ml of 50% nickel-nitrilotriacetic acid (Qiagen) was added to the supernatant and incubated for 1 h at 4°C on a rocking platform. The proteins were washed 6 times with 10 ml of buffer containing 50 mM NaH_2_PO_4_ (pH 8.0), 300 mM NaCl and 50 mM imidazole and eluted with buffer containing 50 mM NaH_2_PO_4_ (pH 8.0), 300 mM NaCl and 250 mM imidazole. After purification, the eluate was exchanged with 10 mM Tris-HCl (pH 7.5)/138 mM NaCl/2.7 mM KCl (Tris-buffered saline (TBS)) and concentrated using Amicon Ultra-15 (MW 10, 000; Millipore). Glycerol was added to the protein solution to a 50% final concentration and stored at -80°C.

**Autokinase Assay to Determine Dependence of PmrB_c_ Autophosphorylation on ATP Concentration.** ATP stock solutions were made by diluting 30 µCi of [γ^32^P] ATP (3000 Ci/mmole) to a final concentration of 50 mM with cold ATP. The His6-PmrB_c_ (5 µM) protein was incubated with a range of ATP concentrations (0.2 µM - 1mM) in 10 µl of TBS/1 mM MgCl_2_/1 mM dithiothreitol (DTT) at room temperature. The reaction was started with addition of [γ^32^P] ATP to the mixture and was stopped after 15 min by mixing with 4x LDS sample buffer (Invitrogen). Samples were kept on ice until they were loaded onto an 8–12% Bis-Tris gel (Invitrogen). After electrophoresis, the gel was autoradiographed and the degree of phosphorylation was determined by quantifying the [^32^P]-labeled species using a BAS-5000 imaging system and phosphor imaging plate (Fuji Film), followed by quantification with Multi Gauge software (Fuji Film). The data were fitted using non-linear regression to the Michaelis-Menten equation (Prism4, Graphpad). Data correspond to mean values of three independent experiments.

**Autokinase Assay to Determine the Rate of PmrB_c_ Autophosphorylation.** The His6-PmrB_c_ (5 µM) protein was incubated with 0.75 mM [γ^32^P] ATP in 120 µl of TBS/1 mM MgCl_2_/1 mM DTT at room temperature. The reaction was started with addition of ATP to the mixture. A 10 µl aliquot was mixed with 4x LDS sample buffer (Invitrogen) at different time points to stop the reaction. Samples were kept on ice until they were loaded onto an 8–12% Bis-Tris gel (Invitrogen). After electrophoresis, the gel was autoradiographed and the degree of phosphorylation was determined by quantifying the [^32^P]-labeled species using a BAS-5000 imaging system and phosphor imaging plate (Fuji Film) followed by quantification with Multi Gauge software (Fuji Film). The data were fitted using nonlinear regression to a one-phase exponential association (Prism4, Graphpad). Data correspond to mean values of three independent experiments.

**Phosphotransferase Assay.** Phosphorylated His6-PmrB_c_ proteins were generated by incubating 10 µM His6-PmrB_c_ with 0.75 mM [γ^32^P] ATP in TBS/1 mM MgCl_2_/1 mM DTT at room temperature for 1 h. Excess [γ^32^P] ATP was removed using a Micro Bio-Spin 6 Chromatography Column (Biorad) that had been preequilibrated with TBS. 5 µl of the mixture was kept as reference samples for the PmrB_c_-P protein before and after the phosphotransfer reaction. To start the phosphotransfer reaction, an equivalent volume of His6-PmrB_c_–P protein mixture was added to 20 µM PmrA-His6 in TBS/1 mM MgCl_2_/1 mM DTT and incubated at room temperature. A 10 µl aliquot was mixed with 4x LDS sample buffer (Invitrogen) at different time points to stop the reaction. Samples were kept on ice until SDS-PAGE. After electrophoresis, the gel was autoradiographed and the degree of phosphorylation was determined by quantifying the [^32^P]-labeled species using a BAS-5000 imaging system and phosphor imaging plate (Fuji Film) followed by quantification with Multi Gauge software (Fuji Film). Data correspond to mean values of three independent experiments.

**Phosphatase Assay.** Phosphorylated PmrA-His6 proteins were generated using phosphorylated GST-PmrB_c_T156R beads as described [4]. Phosphorylated PmrA-His6 was incubated with 5 µM His6-PmrB_c_ protein in TBS/1 mM MgCl_2_/1 mM DTT at room temperature. A 10 µl aliquot was mixed with 4x LDS sample buffer (Invitrogen) at different time points to stop the reaction. Samples were kept on ice until SDS-PAGE. After electrophoresis, the gel was autoradiographed and the degree of phosphorylation was determined by quantifying the [^32^P]-labeled species using a BAS-5000 imaging system and phosphor imaging plate (Fuji Film), followed by quantification with Multi Gauge software (Fuji Film). The data were fitted using nonlinear regression to a one-phase exponential decay (Prism4, Graphpad). Data correspond to mean values of three independent experiments.

**Sequencing of the *pmrB* gene in *Salmonella* and *E. coli* Natural Isolates.** The *pmrB* gene was amplified with AccuPrime^TM^ TaqDNA Polymerase High Fidelity (Invitrogen) using primers upstream and downstream the *pmrB* ORF with the following primer pairs: *Salmonella pmrB*, 1984 and 9842; and *E. coli* *pmrB*, 9840 and 9841. PCR products were purified with the QIAquick PCR purification kit (Qiagen). Sequencing reactions were initiated with the following primers: *Salmonella pmrB*, 1984 and 9842; and *E. coli* *pmrB*, 9840 and 9841, performed using BIG DYE 3.1 (Applied Biosystems) and analyzed on a 3130 Genetic Analyzer (Applied Biosystems). DNA sequences were translated using Vector NTI software (Invitrogen). The *pmrB* gene sequences have been deposited at GenBank under the accession numbers listed in Table S4.

**McDonald-Kreitman Test.** The McDonald-Kreitman test was performed for the *pmrB* sequence data using DNASP v 5.0 [5].

**References**

1. Datsenko KA, Wanner BL (2000) One-step inactivation of chromosomal genes in *Escherichia coli* K-12 using PCR products. Proc Natl Acad Sci U S A 97: 6640-6645.

2. Kato A, Latifi T, Groisman EA (2003) Closing the loop: the PmrA/PmrB two-component system negatively controls expression of its posttranscriptional activator PmrD. Proc Natl Acad Sci U S A 100: 4706-4711.

3. Fields PI, Swanson RV, Haidaris CG, Heffron F (1986) Mutants of *Salmonella typhimurium* that cannot survive within the macrophage are avirulent. Proc Natl Acad Sci U S A 83: 5189-5193.

4. Kato A, Groisman EA (2004) Connecting two-component regulatory systems by a protein that protects a response regulator from dephosphorylation by its cognate sensor. Genes Dev 18: 2302-2313.

5. Librado P, Rozas J (2009) DnaSP v5: a software for comprehensive analysis of DNA polymorphism data. Bioinformatics 25: 1451-1452.
